# Juvenile Hormone Membrane Signaling Enhances its Intracellular Signaling Through Phosphorylation of Met and Hsp83

**DOI:** 10.3389/fphys.2022.872889

**Published:** 2022-04-27

**Authors:** Yue Gao, Nan Chen, Xiangle Zhang, Emma Y. Li, Wei Luo, Jie Zhang, Wenqiang Zhang, Sheng Li, Jian Wang, Suning Liu

**Affiliations:** ^1^ Guangdong Provincial Key Laboratory of Insect Developmental Biology and Applied Technology, Institute of Insect Science and Technology, School of Life Sciences, South China Normal University, Guangzhou, China; ^2^ International Department, The Affiliated High School of South China Normal University, Guangzhou, China; ^3^ Guangmeiyuan R&D Center, Guangdong Provincial Key Laboratory of Insect Developmental Biology and Applied Technology, South China Normal University, Meizhou, China; ^4^ Department of Entomology, University of Maryland, College Park, MD, United States

**Keywords:** phosphoproteomics, proteomics, phosphorylation, Met/Gce, biotinylation, 14-3-3 proteins, hsp83

## Abstract

Juvenile hormone (JH) regulates insect development and reproduction through both intracellular and membrane signaling, and the two pathways might crosstalk with each other. Recent studies have reported that JH membrane signaling induces phosphorylation of the JH intracellular receptor Met, thus enhancing its transcriptional activity. To gain more insights into JH-induced Met phosphorylation, we here performed phosphoproteomics to identify potential phosphorylation sites of Met and its paralog Germ-cell expressed (Gce) in *Drosophila* Kc cells. *In vitro* experiments demonstrate that JH-induced phosphorylation sites in the basic helix-loop-helix (bHLH) domain, but not in the Per-Arnt-Sim-B (PAS-B) domain, are required for maximization of Met transcriptional activity. Moreover, phosphoproteomics analysis reveale that JH also induces the phosphorylation of Hsp83, a chaperone protein involved in JH-activated Met nuclear import. The JH-induced Hsp83 phosphorylation at S219 facilitates Hsp83-Met binding, thus promoting Met nuclear import and its transcription. By using proteomics, subcellular distribution, and co-immunoprecipitation approaches, we further characterized 14-3-3 proteins as negative regulators of Met nuclear import through physical interaction with Hsp83. These results show that JH membrane signaling induces phosphorylation of the key components in JH intracellular signaling, such as Met and Hsp83, and consequently facilitating JH intracellular signaling.

## 1 Introduction

Juvenile hormone (JH), which is primarily produced and secreted by the corpus allatum (CA) in insects, is crucial for regulating metamorphosis and reproduction. Methoprene-tolerant (Met), a transcription factor belonging to the basic helix-loop-helix (bHLH)/Per-Arnt-Sim (PAS) family, was first identified in *Drosophila melanogaster* (Wilson and Fabian, 1986). However, the absence of obvious developmental defects in *Met* mutant argues against the function of Met as a genuine JH receptor (Wilson and Fabian, 1986; Ashok et al., 1998). A paralog of Met, Germ-cell expressed (Gce), takes over the functions of Met in JH action due to functional redundancy (Baumann et al., 2010; Abdou et al., 2011). Both Met and Gce exhibit high affinity for JH *in vitro* (Miura et al., 2005; Charles et al., 2011). Importantly, *Met Gce* double mutant dies during the larval-pupal transition (Abdou et al., 2011), exhibiting a phenotype resembling those of JH-deficient insects (Liu et al., 2009; Riddiford et al., 2010). The requirement of direct hormone binding to Met and Gce *in vivo* for JH-induced downstream gene expression and normal development strongly supports Met and Gce being as JH receptors in *Drosophila* (Jindra et al., 2015b). In the absence of JH or its analog, Met forms a homodimer or heterodimer with Gce, but the formation of this dimer decreases drastically upon binding to JH (Godlewski et al., 2006). Then, Met binds to another bHLH-PAS protein, Taiman (Tai), also known as steroid receptor coactivator (SRC), in a JH-dependent manner (Charles et al., 2011; Li et al., 2011; Zhang et al., 2011; Kayukawa et al., 2012). The chaperone heat shock protein 83 (Hsp83) facilitates nuclear import of Met by physically interacting with its PAS-B and bHLH domains and thus activates the expression of the JH primary response gene *Krüppel homolog 1* (*Kr-h1*) (He et al., 2014). Nucleoporin 358 kD (Nup358) also promotes JH-induced Met nuclear transport dependent on importin β and Hsp83 (He et al., 2017).

In addition to the above JH intracellular pathway, a hypothetical JH membrane signaling has been characterized in a number of insects (Davey, 2000). This rapid and reversible hormonal response initiates the phospholipase C (PLC)-protein kinase C (PKC) pathway and regulates many cellular processes, including “patency” and protein synthesis in insect reproductive organs (Davey and Huebner, 1974; Yamamoto et al., 1988; Bai and Palli, 2016). Both *in vivo* and *in vitro* experiments indicate that JH membrane signaling is able to activate RTK (receptor tyrosine kinase, as the potential JH membrane receptor in Diptera insects)-PLC-PKC pathway independent on JH intracellular signaling (Liu et al., 2015; Gao et al., 2021). On the other hand, previous studies have revealed crosstalk between JH membrane signaling and JH intracellular signaling through phosphorylation of Met and Tai, which modifies the DNA-binding activity of Met/Tai and thus facilitates JH intracellular signaling (Liu et al., 2015; Ojani et al., 2016). Protein phosphorylation, a key posttranslational modification, commonly occurs in various cellular signaling pathways involving subcellular localization, DNA binding and protein-protein interactions with target transcription factors (Hunter and Karin, 1992). Met and other JH signaling components are phosphoproteins (Liu et al., 2015; Kim et al., 2021; Li et al., 2021). Phosphorylation of JH signaling components through a rapid mode of second-messenger signaling might modulate JH action (Jindra et al., 2015a). In *A. aegypti*, calcium/calmodulin-dependent protein kinase II (CaMKII) is proposed to phosphorylate Met and Tai, and enhance Met-Tai DNA binding activity with JH response elements (JHRE) (Liu et al., 2015). Additionally, JH exposure induces dephosphorylation of Kr-h1 and promotes its transcriptional activity (Kim et al., 2021). In *Helicoverpa armigera*, phosphorylation of Met1 in the PAS-B domain is important for binding of Met1 to JHRE in the *Kr-h1* promoter and increases interaction of Met1 with Tai (Li et al., 2021). However, the underlying mechanisms of Met phosphorylation and interaction between JH membrane and intracellular signaling are still not completely understood.

To address these issues, we detected multiple phosphorylation sites of Met and Gce after methoprene treatment with a biotinylation tagging method and found that phosphorylation sites in the bHLH domain are important for transcriptional activity of Met. Second, JH membrane pathway phosphorylated Hsp83 at S219 to promote Met nuclear import and enhance JH intracellular signaling. Finally, 14-3-3 proteins recognized Hsp83 that seemed acting as a bridge between Met/Gce and 14-3-3 proteins, and subsequently sequester Met in the cytoplasm to inhibit its nuclear import. This work provides novel insights into JH signaling transduction in *Drosophila* and would be helpful to advance our understanding of the complex JH signaling network.

## 2 Materials and Methods

### 2.1 DNA Constructs

The full-length open reading frame of *Drosophila Met* (NM_078571) and *Gce* (NM_078605) open reading frame was amplified from the larval fat body cDNA library, and *BirA* (NC_000913.3) was amplified from *E. coli*. All the PCR products were cloned into the pClone007 vector (007VS, TsingKe Biotech). *BirA* was then inserted into the pUAST vector between the EcoRI and XbaI sites to generate a control construct expressing only the BirA protein. For the generation of *Met* and *Gce* overexpression lines, vectors were constructed with the Gibson assembly method. First, the pUAST vector was digested by EcoRI/XbaI. The *P2A-BirA* sequence was amplified from the pClone007-BirA vector. When a P2A peptide (ATNFSLLKQAGDVEENPGP) was added to the N-terminus of BirA, 5 amino acids (GGSGS) were added to the N-terminus, and 3 amino acids (GGS) were added to the C-terminus of the P2A peptide as a linker. The FLAG tag (DYKDDDDK) and an Avi tag (GLNDIFEAQKIEWHE) were added to the 5′ end by reamplifying the above PCR products with FLAG-Avi-P2A-F oligos and the BirA-R oligo. PCR of the coding sequence of *Met* or *Gce* from the pClone007-Met or pClone007-Gce vector was performed, and then we used a Hieff Clone Plus Multi One Step Cloning Kit (10912ES10, Yeasen) for assembly. Finally, we obtained the *pUAST-Met-4 × FLAG-Avi-P2A-BirA* (*UAS-Met-BirA*) or *pUAST-Gce-3 × FLAG-Avi-P2A-BirA* (*UAS-Gce-BirA*) vector, in which BirA was fused at the C-terminus. Meanwhile, we also constructed *pUAST-BirA-P2A-Met-3 × FLAG-Avi* and *pUAST-BirA-P2A-Gce-3 × FLAG-Avi*, with BirA fused at the N-terminus, using a similar method to assess the translational efficiency of different termini.

Moreover, the full-length cDNAs of *Met*, *Gce*, *Hsp83* (NP_523899), *14-3-3ε* (NM_169796) and *14-3-3ζ* (NM_001273916) were amplified. Met-FLAG, Met-bHLH+(amino acids 1–97)-FLAG, Met-PAS-A+(amino acids 88–196)-FLAG, Met-PAS-B+(amino acids 187–716)-FLAG, Met-PAS-B (amino acids 403–510)-FLAG, Met-PAS-B + ^T265A^-FLAG, Met-PAS-B + ^S531A^-FLAG, Met-PAS-B + ^T535A^-FLAG, Met-PAS-B + ^S652A^-FLAG, Met-PAS-B + ^T265A/S531A/T535A/S652A^-FLAG (4 m), Met-4m-FLAG, Met^S256A/T265A/S531A/T535A/S651A/S652A^-FLAG (6 m), Gce-FLAG, Gce-bHLH+(amino acids 1–335)-FLAG, Gce-PAS-A+(amino acids 326–415)-FLAG, Gce-PAS-B+(amino acids 406–956)-FLAG, Hsp83-FLAG, Hsp83-V5, Hsp83^S219A^-V5, 14-3-3ε-HA, and 14-3-3ζ-HA were separately generated and constructed into the pUAST vector for overexpression in Kc cells.

### 2.2 Identification of Phosphorylation Sites of Met/Gce and Proteomics of Their Conjugates

#### 2.2.1 Sample Collection

*Drosophila* Kc cells were cultured in Schneider’s medium. *UAS-BirA*, *UAS-Met-BirA*, *UAS-Gce-BirA* or both was co-transfected with the *Act5c-Gal4* vector into cells using Effectene transfection reagents (#301427, QIAGEN) according to the manufacturer’s instructions. After 47 h, the old medium was replaced with fresh medium containing 10 μM methoprene (#16807, Cayman Chemical Company) and then incubated for an additional 1 h. The harvested cells were lysed in ice-cold NP-40 lysis buffer (P0013, Beyotime) supplemented with protease and phosphatase inhibitor cocktail (P1045, Byotime) and protease inhibitor cocktail (#87785, Thermo Fisher Scientific) for western blotting and immunoprecipitation. Immunoprecipitation was performed overnight at 4°C with streptavidin beads (#888010, Fitgene) and was followed by three 15 min washes using lysis buffer supplemented with protease and phosphatase inhibitor cocktail and protease inhibitor cocktail. After washing, the purified proteins were eluted by competitive elution and boiling elution. Competitive elution was eluted with free biotin or with an equal bead volume of SDS sample buffer containing dithiothreitol (DTT) at 95°C for 5 min, boiling elution was eluted by boiling bead after adding protein loading buffer. For phosphorylation site mapping, Met or Gce proteins were resolved by 10% SDS-PAGE, and the excised bands were used for protease digestion and mass spectrometry.

#### 2.2.2 LC-MS/MS Analysis

After protease digestion, peptides were dissolved in 0.1% formic acid (FA) and 2% acetonitrile (ACN) and separated by a reversed-phase analytical column (15 m × 75 μm, packed with Acclaim PepMap C18, 2 μm, 100 A, Thermo Fisher Scientific). LC separation of the peptides was initiated with an increasing gradient from 5 to 50% solvent B (0.1% FA in 80% ACN) over 20 min, and ramped to 90% for 10 min, followed by a 5 min holding. All steps were performed at a constant flow of 300 nL/min. MS analysis was performed on an Orbitrap Fusion Lumos Tribrid mass spectrometer (Thermo Fisher Scientific). Intact peptides were detected in the Orbitrap at a resolution of 70,000. The peptides were selected for MS/MS using a normalized collision energy (NCE) setting of 27, and ion fragments were detected in the Orbitrap at a resolution of 17,500. A data-dependent procedure that alternated between one MS scan followed by 20 MS/MS scans was applied for the top 20 precursor ions above a threshold ion count of 1E4 in the MS survey scan with 30 s dynamic exclusion. The electrospray voltage applied was 2.0 kV. Automatic gain control (AGC) was used to prevent overfilling of the ion trap; 1E5 ions were accumulated for generation of MS/MS spectra. For MS scans, the scan range was 350–1800 m/z.

#### 2.2.3 Data Analysis

Protein identification was performed with MASCOT software by searching the UniProt *D. melanogaster* Reference Sequences database (21,933 proteins, 6/2018). The parameters were as follows: trypsin (full), 2 maximum missed cleavages, cysteine carbamidomethylation (C) as the fixed modification, and methionine oxidation as the variable modification. For phosphorylation site mapping, phosphorylation of serine/threonine/tyrosine was added as another variable modification. The precursor ion mass tolerance in the initial search was 20 ppm. The results were filtered according to a 1% false discovery rate (FDR) at the peptide and protein levels.

### 2.3 Identification of Phosphorylation Sites of Hsp83

#### 2.3.1 Sample Collection

*UAS-Hsp83-V5* was co-transfected with the *Act5c-Gal4* vector into cells using Effectene transfection reagents according to the manufacturer’s instructions. After 46 h, the old medium was replaced with fresh medium containing 10 μM methoprene, and the cells were incubated for an additional 2 h. The harvested cells were lysed in ice-cold NP-40 lysis buffer (supplemented with protease and phosphatase inhibitor cocktail and protease inhibitor cocktail) for immunoprecipitation. The lysates were incubated with V5 antibody for 4 h and then with Pierce protein A/G agarose (#UC277911, Thermo Fisher Scientific) overnight at 4°C. Beads were collected by slow centrifugation, washed 4 times with lysis buffer and resolved by 10% SDS-PAGE. The excised bands were used for protease digestion and mass spectrometry.

#### 2.3.2 LC-MS/MS Analysis

After protease digestion, the peptides were separated in a C18 analytical column (15 m × 150 μm × 1.9 μm). The gradient comprised a decrease from 94 to 0% mobile phase A (0.1% FA) and an increase from 6 to 100% mobile phase B (0.1% FA in 80% ACN) over 25 min and then a hold at 0% mobile phase A and 100% mobile phase B for 5 min. The separated peptides were analyzed with a Q Exactive HF mass spectrometer (Thermo Fisher Scientific). The full scan range was from 350 to 1,500 m/z, the resolution was 60,000, the automatic gain control target value was 3 × 10^6^, and the maximum ion injection time was 20 ms. The top 20 most abundant precursors in the full scan were selected, fragmented by higher-energy collisional dissociation and analyzed *via* MS/MS with a resolution of 15,000, an AGC target value of 1 × 10^5^, a maximum ion injection time of 45 ms, a normalized collision energy of 27%, an intensity threshold of 2.2 × 10^4^, and a dynamic exclusion time of 20 s.

#### 2.3.3 Data Analysis

Protein identification was performed with Proteome Discoverer 2.2 (PD 2.2, Thermo Fisher Scientific) by searching the UniProt *D. melanogaster* Reference Sequences database (42,756 proteins, 8/2020). The search parameters were set as follows: carbamidomethyl was specified in PD 2.2 as a fixed modification, oxidation of methionine and acetylation of the N-terminus were specified in PD 2.2 as variable modifications, serine/threonine/tyrosine phosphorylation was another variable modification, the mass tolerance for precursor ions was 10 ppm and a maximum of 2 missed cleavage sites were allowed. The results were filtered to a 1% FDR at the peptide and protein levels.

### 2.4 Western Blotting

Western blotting was performed as previously described (He et al., 2014; Gao et al., 2021). In brief, cells were lysed on ice in NP-40 lysis buffer supplemented with protease and phosphatase inhibitor cocktail, and the concentration of the total protein was determined with a BCA reagent. An aliquot of 20 μg of the protein extracts was separated by a 10% SDS-PAGE gel and immediately transferred onto a PVDF membrane. The membranes were then blocked with 5% non-fat milk and incubated with 1: 2000 primary antibodies at 4°C overnight, followed by three washes with PBST. The protein bands were finally incubated with 1: 5,000 secondary antibodies and visualized by chemiluminescence. The primary antibodies used in this study were HRP-linked goat anti-biotin (#7075, Cell Signaling Technology, CST), mouse anti-FLAG (#F1804, Sigma-Aldrich), rabbit anti-V5 (#ab9116, Abcam), mouse anti-HA (#sc-7392, Santa Cruz), rabbit anti-phospho-PKC substrate (#2261, CST), rabbit anti-phospho-CaMKII (#12716, CST), and mouse anti-α-tubulin (#AT819, Beyotime) antibodies, and HRP-labeled secondary antibodies (#sc-2313 and sc-2318, Santa Cruz) were used.

### 2.5 Two-Dimensional Gel Electrophoresis

2D gel electrophoresis was performed as previously described (Liu et al., 2015). Total proteins from Kc cells were extracted with NP-40 lysis buffer as described above. The protein extracts from each treatment were precipitated with ice-cold trichloroacetic acid/acetone and were then redissolved in rehydration buffer (8 M urea, 4% 3-[(3-Cholamidopropyl)-dimethyl- ammonio]-1-propane sulfonate [CHAPS], 65 mM DTT, 0.2% ampholytes, and 0.001% bromophenol blue). Equal amounts of the total proteins (150 μg) from each sample were loaded onto an immobilized pH gradient (IPG) strip with the pH ranging from 3 to 10 (Bio-Rad). Isoelectric focusing (IEF) was performed using an Ettan IPGphor 3 IEF System (GE Healthcare) following the manufacturer’s instructions. After IEF, the IPG strips were successively equilibrated in a reducing solution (0.375 M Tris-HCl, pH 8.8, 2% DTT, 6 M urea, 2% SDS, and 20% glycerin) and an alkylating solution (0.375 M Tris-HCl, pH 8.8, 2.5% iodoacetamide, 6 M urea, 2% SDS, 20% glycerin) each with 15 min. The equilibrated strips were then subjected to 2D SDS-PAGE. After electrophoresis, the proteins were transferred to polyvinylidene fluoride (PVDF) membranes for probing with an antibody against biotin.

### 2.6 Coimmunoprecipitation

Forty-six hours following transfection, 10 μM methoprene (stock concentration of 100 μg/μL, dissolved in DMSO) or DMSO was added to Kc cells for 2 h. The cells were then washed with PBS and harvested and lysed in ice-cold NP-40 lysis buffer containing protease and phosphatase inhibitor cocktail and protease inhibitor cocktail. The extracts were clarified by centrifugation at 12000 × *g* for 15 min at 4°C. The lysates were incubated with a FLAG, V5, or HA antibody for 4 h and then with Pierce protein A/G agarose overnight at 4°C. Beads were collected by slow centrifugation, washed 4 times with lysis buffer and analyzed by SDS-PAGE followed by western blotting.

### 2.7 Immunohistochemistry

The subcellular localization of Met-FLAG, Met-bHLH + -FLAG, Met-PAS-A + -FLAG, Met-PAS-B + -FLAG, Met-PAS-B-FLAG, Met-PAS-B + ^T265A^-FLAG, Met-PAS-B + ^S531A^-FLAG, Met-PAS-B + ^T535A^-FLAG, Met-PAS-B + ^S652A^-FLAG Met-PAS-B+^4m^-FLAG, Gce-bHLH + -FLAG, Gce-PAS-A + -FLAG, Gce-PAS-B + -FLAG, Met-4m-FLAG, Met-6m-FLAG, 14-3-3ε-HA, and 14-3-3ζ-HA was measured by immunohistochemistry as described previously (He et al., 2017; Liu et al., 2018). Briefly, the cells were fixed with 4% formaldehyde for 30 min. After three washes with PBT (PBS with 0.3% Triton X-100 and 0.5% bovine serum albumin), the cells were incubated with primary antibodies (1: 1,000) at 4°C overnight followed by three PBT washes. Incubation of the secondary antibody (1: 1,000) was then performed for 2 h at room temperature. The primary antibodies used were rabbit anti-FLAG (#14793, CST, diluted 1: 200) and mouse anti-HA (#sc-7392, Santa Cruz) antibodies. The secondary antibodies used were Alexa Fluor 488-conjugated goat anti-rabbit IgG (#A11008, Invitrogen, 1: 200 diluted) and Alexa Fluor 594-conjugated goat anti-mouse IgG (#A11032, Invitrogen, 1: 200 diluted). The nuclei were stained with DAPI at 1: 2000 (#C1002, Beyotime). Confocal images were collected on an Olympus FluoView FV3000 confocal microscope.

### 2.8 qRT-PCR

Total RNA samples were prepared from Kc cells using RNAiso Plus regents (Takara). All qRT-PCR was performed with at least three biological replicates, each with triplicates, by using Hieff qPCR SYBR Green Master Mix (#11202ES03, Yeasen) on a QuantStudio 6 Flex Real-Time PCR system (Applied Biosystems). The final volume of each reaction was 20 μL, and the PCR thermocycling was initiated from 95°C for 2 min, followed by 40 cycles of 95°C for 10 s, and 60°C for 30 s. After the PCR amplification, a melting program was also included to verify the specificity of the products. Relative expressions of the target genes were calculated by the 2^−ΔΔCT^ method, with *rp49* gene as an internal reference for normalization. All the primers involved are summarized in Supplementary Table S7.

### 2.9 Luciferase Reporter Assays

*pGL3-JHRR* (He et al., 2014) and the reference pRL vector were co-transfected into Kc cells with 14-3-3 protein overexpression vectors. Forty-six hours after transfection, 10 μM methoprene or DMSO was added to Kc cells, and the cells were incubated for an additional 2 h. Dual luciferase assays were conducted using a Dual Luciferase Assay System (Promega) as previously reported (Tian et al., 2013).

### 2.10 Statistics

Unless otherwise specified, all the data subjected to statistics are mean ± standard deviations from independent replicates, and the statistics was analyzed with IBM SPSS Statistics 19.0. Significant differences between two groups and among multi-treatments were determined with the Student’s *t*-test and one-way analysis of variance (ANOVA) followed by Duncan multiple comparisons test, respectively, at the level of *p* < 0.05.

## 3 Results

### 3.1 JH Induces Met and Gce Phosphorylation

**FIGURE 1:**
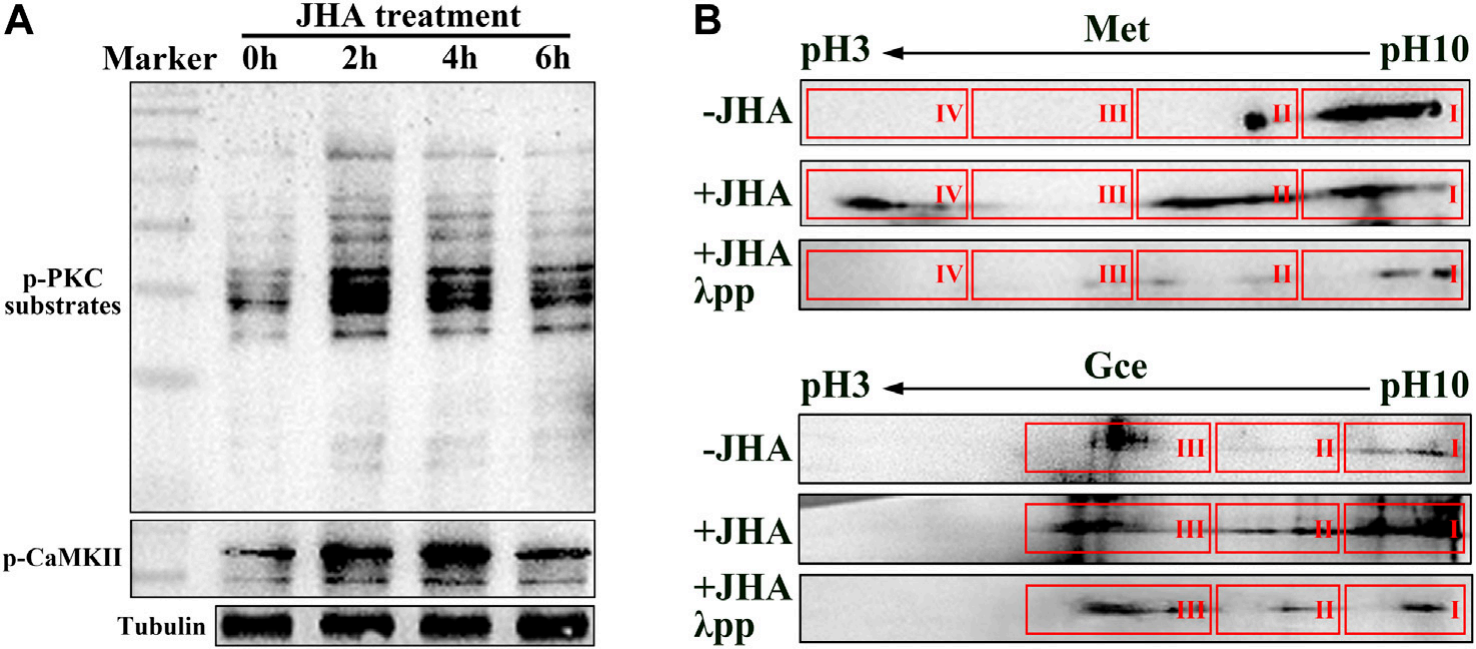
Phosphorylation of Met and Gce in Drosophila Kc cells induced by Methoprene (JHA). **(A)** Phosphorylated PKC substrate and CaMKII levels in response to JHA treatment at various timepoints. The tubulin protein was used as a loading control. **(B)** Validation of phosphorylated Met and Gce by 2D gel electrophoresis followed by western blot analysis. Met and Gce were visualized by antibodies against biotin.

The phosphorylation and transcriptional activity of Met is regulated by JH membrane signaling pathway which involves RTK-mediated phosphorylation of CaMKII and PKC (Liu et al., 2015; Ojani et al., 2016; Gao et al., 2021). We thus first confirmed whether methoprene (JH analog, JHA) induced the phosphorylation of PKC and CaMKII in *Drosophila* Kc cells. In response to JHA, the phosphorylation of both proteins in was elevated at 2–4 h, and the elevation was sustained for 6 h (Figure 1A). By performing 2D gel electrophoresis of the protein extracts followed by western blotting, we detected multiple molecular forms of Met and Gce in a descending order of isoelectric point (pI), and some of these forms migrated to a lower-pH region (Figure 1B). Incubation of JHA-treated protein extracts with λ phosphatase (λpp) before electrophoresis prevented this migration, resulting in a distribution of Met or Gce being similar to that in the control group (Figure 1B). Together with the previous reports (Liu et al., 2015; Ojani et al., 2016; Gao et al., 2021), the above results suggest functional conservation of the PKC and CaMKII in the regulation of phosphorylation of Met and Gce in *Drosophila*.

### 3.2 Isolation and Purification of Met/Gce Proteins With an *in vitro* Biotinylation Method

**FIGURE 2:**
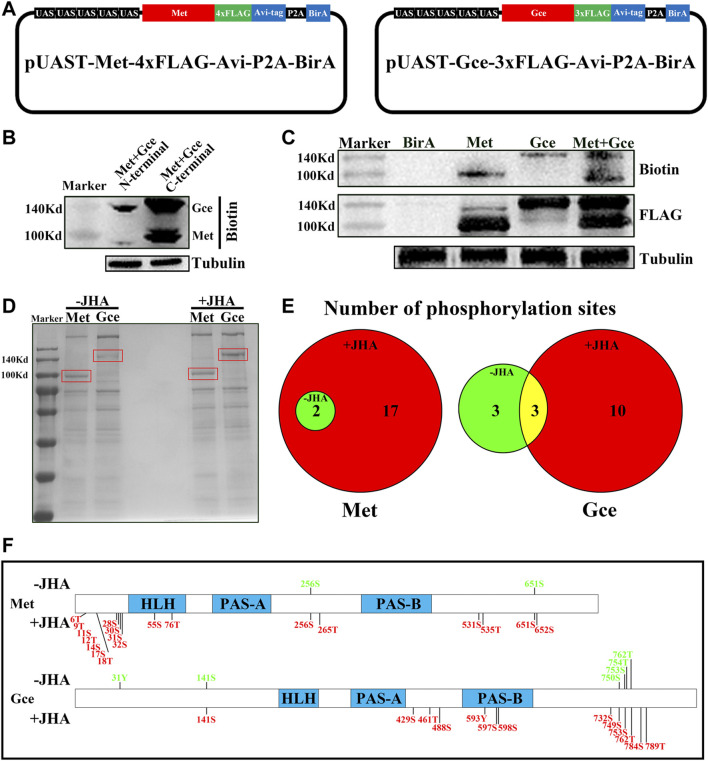
Identification of phosphorylation sites in Met and Gce *in vitro* via a biotinylation tagging approach. **(A)** Schematic diagram of the UAS-Met-BirA and UAS-Gce-BirA constructs for *in vitro* biotinylation. **(B)** Effect of BirA fused to either C-terminus or N-terminus of Met and Gce on the yield of the two proteins. **(C)** Double validation of Met or Gce expression by both biotin and FLAG antibodies. The tubulin protein was used as a loading control. **(D)** Separation of phosphorylated proteins by coomassie blue staining after immunoprecipitation of the protein extracts from Met-BirA or Gce-BirA overexpressing cells. Met and Gce bands (in red frame) were excised for protease digestion and mass spectrometry. **(E)** Venn diagram showing the numbers of phosphorylation sites that were identified for either Met or Gce, with regard to JHA treatment. **(F)** Distribution of the identified phosphorylation sites in Met and Gce. The sites in green indicate basal phosphorylation sites (2 for Met and 3 for Gce) in consistent with those in **(E)**.

To isolate and purify Met/Gce, we used an *in vitro* biotinylation strategy and then examined the phosphorylation profile of Met/Gce by liquid chromatography-tandem mass spectrometry (LC-MS/MS). We generated vectors that express *Met-BirA* or *Gce-BirA*, a synthetic linear construct with an added biotin ligase (BirA) molecule at the C-terminus (Figure 2A). Each target protein in the construct had a FLAG and biotin acceptor peptide (Avi) tag that could be effectively biotinylated by the *Escherichia coli* BirA enzyme (Beckett et al., 1999; de Boer et al., 2003). We also included a porcine teschovirus-1 2A (P2A) self-cleaving peptide that shows high efficiency in facilitating the expression of multiple transgenes in *Drosophila* (Daniels et al., 2014; Liu et al., 2017). These constructs as well as a control construct with only the BirA were expressed in Kc cells using the *Act5c-Gal4*. The protein-biotin strategy was first used to purify JH intracellular receptors and their conjugates in either JHA- or dimethyl sulfoxide (DMSO)-treated cells. To assess the translational efficiency of different termini, we initially tested Met or Gce protein levels in Kc cells. We found that Met and Gce protein levels were higher when BirA was fused at the C-terminus than that at the N-terminus (Figure 2B). For consistency, *UAS-Met-BirA* and *UAS-Gce-BirA* were used in all subsequent studies. Both types of cells expressing Met and Gce, which all included biotin and FLAG, showed bands of the same size (Figure 2C), indicating that this approach was viable for our purpose.

### 3.3 Identification of Met or Gce Phosphorylation Sites

To identify the phosphorylation sites, Met and Gce proteins were separated by 10% SDS-PAGE, and the target bands were subjected to protease digestion and MS analysis (Figure 2D). A total of 19 phosphorylation sites of Met were identified from the JHA treatment, two of which were also present in the control DMSO treatment. We identified 6 and 13 phosphorylation sites of Gce in the control and JHA treatment groups, respectively; three sites were shared by both groups (Figure 2E; Supplementary Tables S1–S3), indicating that these sites may be basal phosphorylation sites. Moreover, the identified phosphorylation sites of Met were rich in its N-terminus, whereas most of the phosphorylation sites of Gce were distributed in the C-terminus (Figure 2F). This finding suggests that phosphorylation sites in Met and Gce may differ in their molecular functions.

### 3.4 Phosphorylation Sites in Met bHLH Domain are Necessary for JH Intracellular Signaling

**FIGURE 3:**
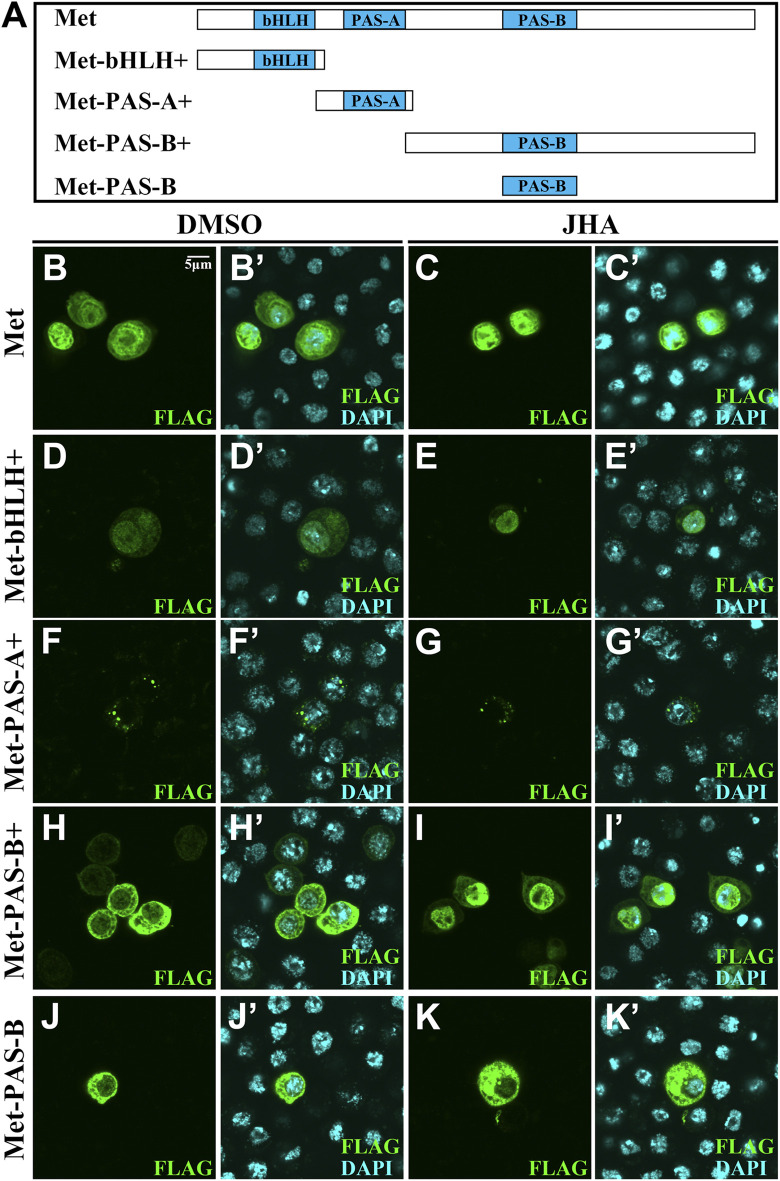
Subcellular localization of distinct fragments of Met with a FLAG tag in Kc cells. **(A)** Scheme illustrating the full-length and truncated mutants of Met. **(B–K’)** Localization of full-length or truncated mutants of Met was not altered in the absence or presence of JHA except for Met-PAS-B+. Cells expressing full-length or truncated mutants of Met were stained for FLAG (green). Merge images also present nuclear staining with DAPI (blue).

The nuclear localization of Met depends on the PAS-B domain in the presence of JH (Greb-Markiewicz et al., 2011). We therefore divided the full-length *Met* into three distinct fragments corresponding to conserved domains predicted by the Conserved Domain Database, including bHLH+ (amino acids 1–97), PAS-A+ (amino acids 88–196) and PAS-B+ (amino acids 187–716) (Figure 3A). Compared to the control, JHA dramatically induced subcellular trafficking of the PAS-B+ part (Figures 3B–I’). By contrast, PAS-B lacked surrounding amino acids was exclusively localized to the cytoplasm (Figures 3J–K’). Likewise, each of the three Gce fragments remained a stable subcellular localization after JH treatment (Supplementary Figure S1). Given the known function of phosphorylation modification in controlling the subcellular localization of transcription factors, these observations suggest that the region surrounding the PAS-B domain of Met that contains phosphorylation sites might affect the subcellular localization of Met.

**FIGURE 4:**
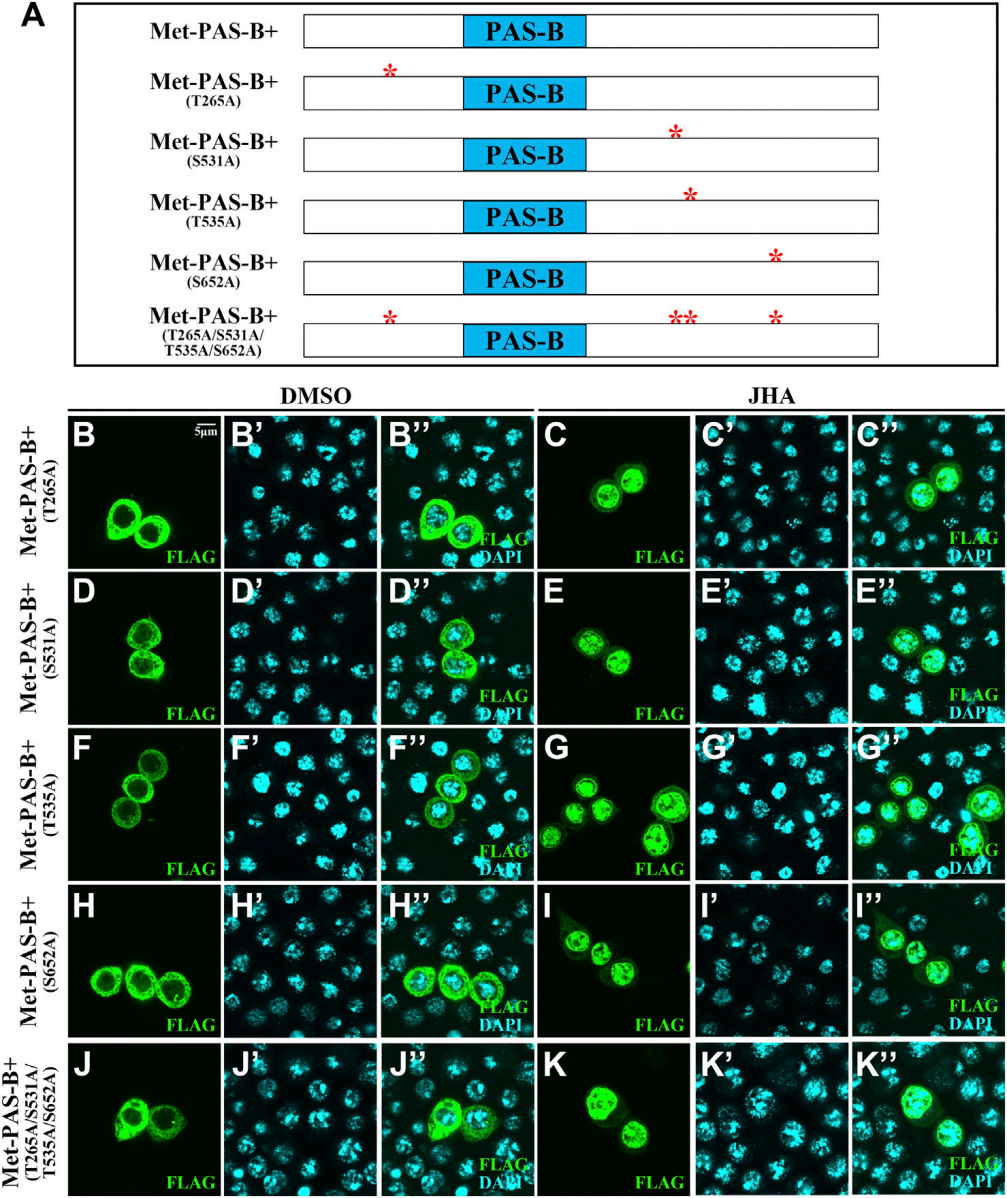
Localization of mutations of phosphorylation site mutants in the PAS-B+ region when Kc cells were treated with DMSO or JHA. **(A)** Scheme illustrating Met-PAS-B+ fragment and mutations of phosphorylation sites in PAS-B+ of Met. **(B–K’’)** Localization of mutations of phosphorylation sites in PAS-B+ of Met was all altered in the presence of JHA compared to the control DMSO treatment. Cells expressing mutations of phosphorylation sites in PAS-B+ of Met were stained for FLAG (green) and DAPI (blue).

**FIGURE 5:**
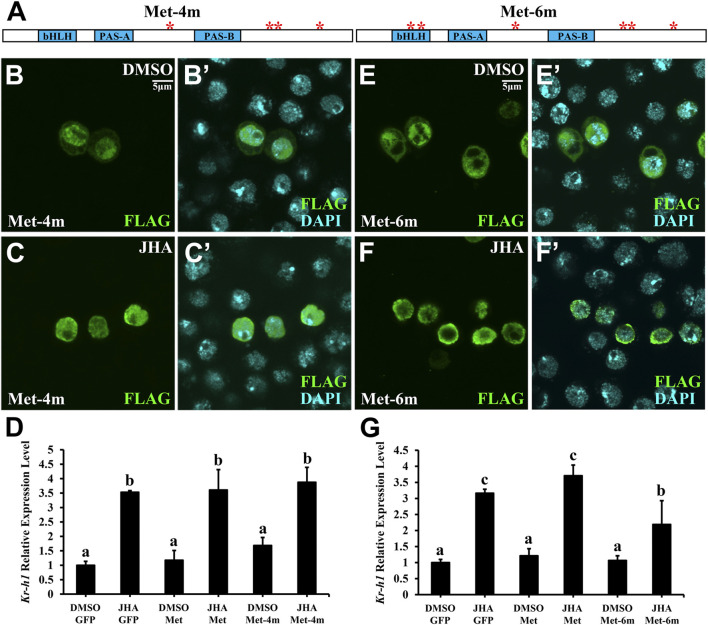
Phosphorylation sites in the bHLH but not the PAS-B domain promote JH signaling activity. **(A)** A schematic diagram showing the mutations of the Thr and Ser residues in Met. **(B–C’)** Subcellular localization of Met-4m in Kc cells treated with JHA for 1 h, compared with that in the DMSO control. **(D)** Effect of GFP, Met or Met-4m overexpression in Kc cells on Kr-h1 mRNA level at 2 h after a 10 μM JHA treatment. **(E–F’)** Subcellular localization of Met-6m in Kc cells treated with JHA for 1 h, compared with that in the DMSO control. **(G)** Effect of GFP, Met or Met-6m overexpression in Kc cells on Kr-h1 mRNA level at 2 h after a 10 μM JHA treatment. For both panels **(D)** and **(G)**, different letters indicate significant differences using one-way ANOVA followed by Duncan multiple comparisons test.

For Met, there were 6 phosphorylation sites, including 2 basal phosphorylation sites. To determine whether these phosphorylation sites in the PAS-B+ region of Met are essential for JH-induced subcellular trafficking, we mutated either phosphorylated serine (S) or threonine (T) to alanine (A) and generated 5 phosphor-inactivated PAS-B+ mutant constructs. All these mutant forms failed to prevent their subcellular trafficking in the presence of JHA (Figure 4). Likewise, even if all 4 phosphorylation sites were mutated in full-length Met (Met-4m), nuclear localization was maintained (Figures 5A–C’). These findings indicate that the phosphorylation sites surrounding the PAS-B domain of Met are dispensable for nuclear import of Met. Moreover, the *Kr-h1* transcriptional level was similar to that in intact Met-overexpressing cells after JHA exposure (Figure 5D).

Since the bHLH domain is responsible for DNA binding (Li et al., 2006), two JH-induced phosphorylation sites (S55 and T76) in the bHLH domain were mutated (Met-6m) on the basis of Met-4m (Figure 5A). Consistent with the findings for Met-4m, mutation of 6 sites caused the protein to be present primarily in the nucleus, regardless of the presence or absence of JHA (Figures 5E–F’). The *Kr-h1* transcriptional level was significantly higher in Met-overexpressing cells treated with JH than in Met-6m-overexpressing cells (Figure 5G). These results suggest that phosphorylation sites in the bHLH domain of Met are necessary for JH intracellular signaling activity.

### 3.5 Identification of Potential Met/Gce Partner Proteins for Regulating Nuclear Import

**FIGURE 6:**
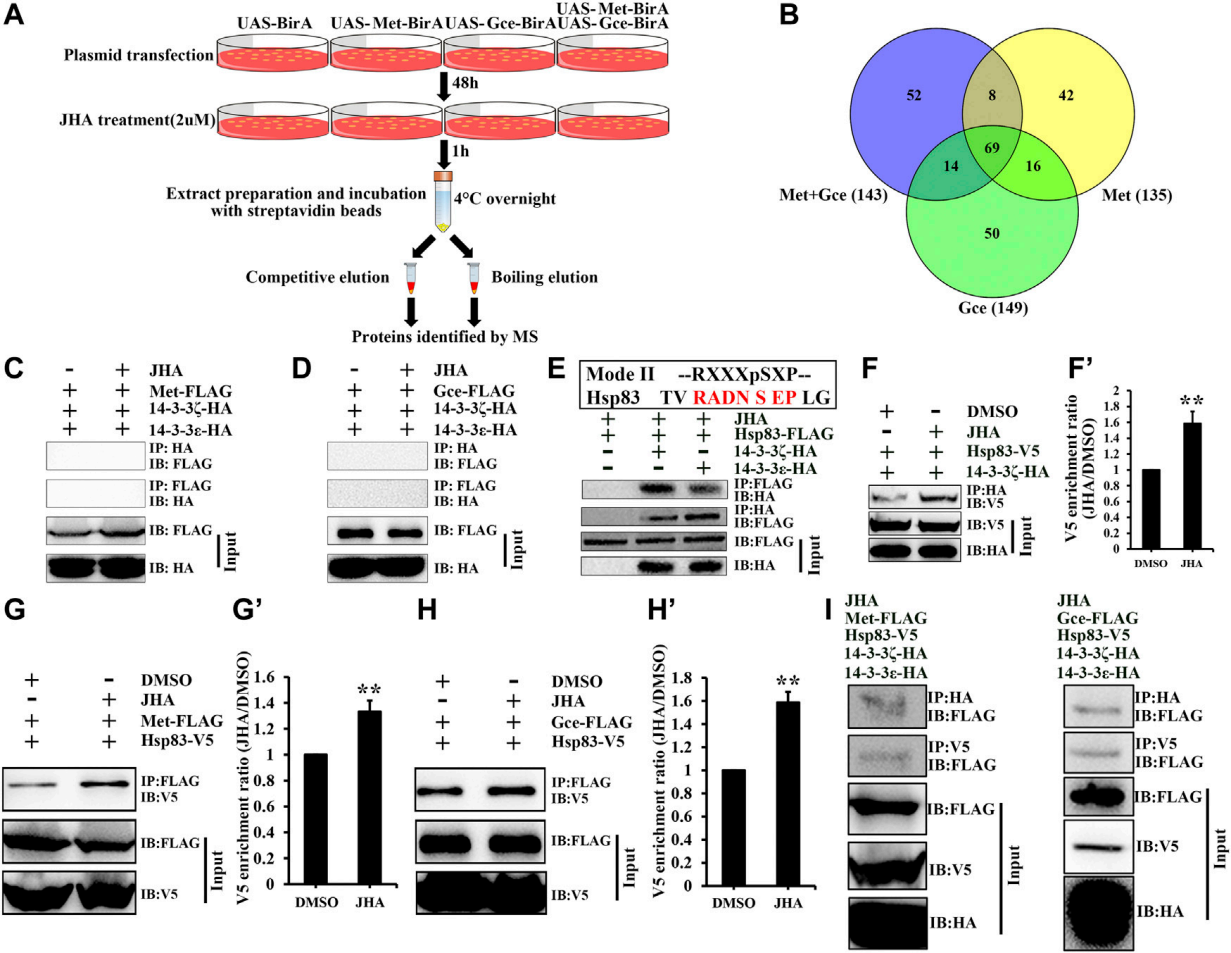
Hsp83 bridges an interaction between Met/Gce and 14-3-3 proteins. **(A)** A flow chart showing the strategy for purification of potential binding proteins of Met/Gce from Drosophila Kc cells. **(B)** Venn diagram showing the numbers of binding protein for Met, Gce or both of them in the presence of JHA. A total of 69 conjugates of Met/Gce were identified in all data sets. See detailed information of the identified proteins in Supplementary Table S5. **(C–E)** 14-3-3 proteins physically interact with Hsp83 **(E)** but not Met **(C)** and Gce **(D)**, as determined by Co-IP. **(F–H’)** JH promotes Hsp83 binding activity with 14-3-3 proteins or Met/Gce. Qualification of V5 **(F’,G’,H’)** of the western blotting results in **(F,G,H)** using tubulin as a control. **p < 0.01 (Student’s t-test). **(I)** Hsp83 interactes with Met/Gce and 14-3-3 to form a tripolymer complex in the presence of JHA. Cell lysates were subjected to immunoprecipitation (IP) with an anti-FLAG **(C,D,E,G,H)**, an anti-HA **(F,I)** and an anti-V5 **(I)**, antibody, respectively. The interacting proteins were detected on immunoblot (IB).

In addition to posttranslational modifications, partner proteins of the Met/Gce complex, such as Tai, ftz transcription factor 1 (Ftz-f1), and Hsp83, are indispensable for modulating JH action or Met nuclear import (Charles et al., 2011; Bernardo and Dubrovsky, 2012; He et al., 2014). To determine potential Met/Gce binding proteins involved in regulating the subcellular localization of JH intracellular receptors, we expressed Avi-tagged Met, Gce, or both constructs within Kc cells and performed pulldown assays with streptavidin beads and LC-MS/MS analysis (Figure 6A). Datasets from competitive elution and boiling elution were pooled for bioinformatics analysis. After deducting the peptides identified from the BirA control, we obtained 135, 149, and 143 peptides for Met expression, Gce expression and Met/Gce co-expression in Kc cells (Figure 6B; Supplementary Table S4); 69 of these peptides were common to all treatments (Figure 6B; Supplementary Table S5). Notably, two 14-3-3 protein isoforms, 14-3-3ε and 14-3-3ζ, were identified in all these examined samples. 14-3-3 proteins often act redundantly in many cellular processes involved in the regulation of subcellular localization (Benton and St Johnston, 2003; Acevedo et al., 2007). They form homo- and heterodimers and participate in many cellular processes, including signal transduction, by binding to specific phosphorylated sites on their target partners (Mackintosh, 2004; Morrison, 2009). These findings suggest that 14-3-3 proteins might affect the subcellular distribution of Met or Gce.

### 3.6 14-3-3 Proteins Antagonize Met Nuclear Import by Physical Interaction With Hsp83

While 14-3-3 proteins have mostly been reported to specifically interact with phosphoserine-containing motifs such as RSXpSXP (mode I) and RXXXpSXP (mode II) in their ligands (Dougherty and Morrison, 2004), some non-consensus phosphorylation and non-phosphorylation motifs in a set of target proteins of 14-3-3 have been reported (Rajan et al., 2002; Ottmann et al., 2007; Ji and Ostap, 2020). However, upon analyzing the amino acid sequences of Met and Gce, we did not find any consensus or putative 14-3-3 binding phosphorylation motifs, implying that 14-3-3 proteins may interact with Met or Gce in a phosphorylation-independent manner. Moreover, neither Met nor Gce was able to coimmunoprecipitate with 14-3-3ε or 14-3-3ζ after DMSO or JHA treatment (Figures 6C, D). These data suggest that 14-3-3 proteins are unable to directly bind to JH intracellular receptors.

We next asked whether there exists a protein(s) acting as molecular bridge between Met/Gce and 14-3-3 proteins. As an important partner protein of Met, Hsp83 is required for Met nuclear import (He et al., 2014). We therefore analyzed the Hsp83 amino acid sequence and identified a putative 14-3-3 binding motif, RADNSEP, which was located at amino acids 161–167 (Figure 6E). Indeed, Hsp83 coimmunoprecipitated with 14-3-3ε or 14-3-3ζ in the absence or presence of JHA, respectively (Figures 6E, F), and the binding affinity of 14-3-3ζ for Hsp83 was dramatically increased by JHA treatment (Figures 6F, F’). Consistent with previous reports that JH promotes the binding affinity of Hsp83 to Met/Gce (Figures 6G–H’) (He et al., 2014), we further showed that in the presence of JHA, Hsp83 interacted with Met/Gce and 14-3-3 to form a tripolymer complex (Figure 6I).

**FIGURE 7:**
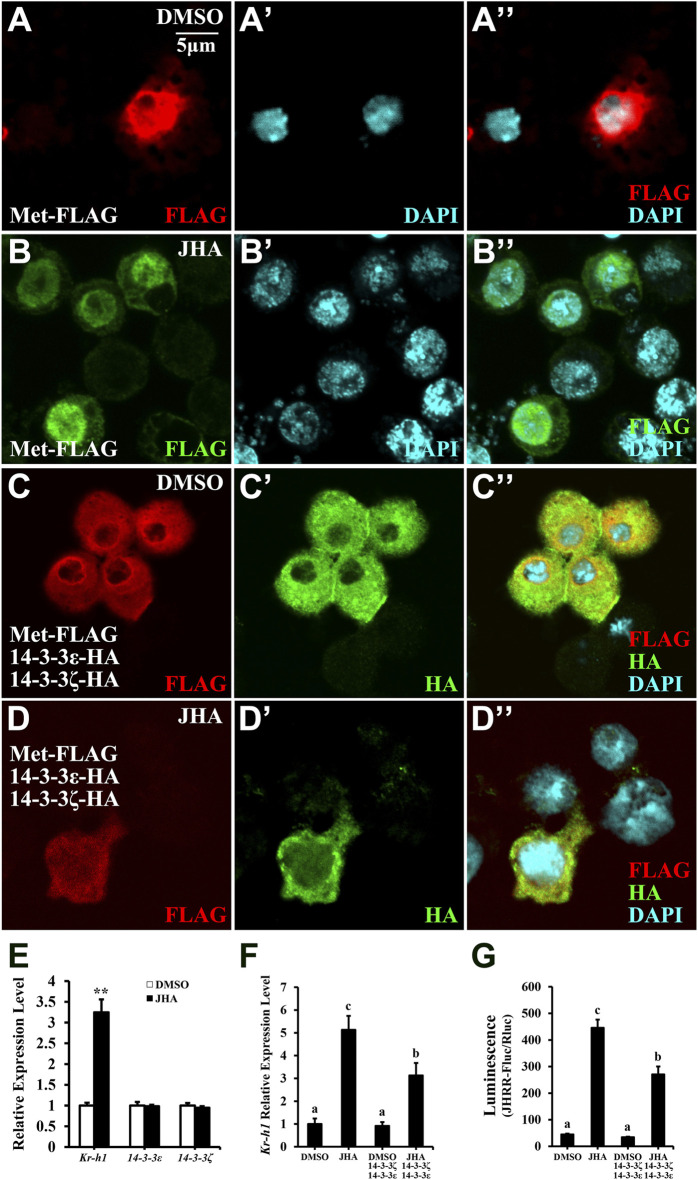
14-3-3 proteins suppress JH signaling activity via sequestrating Met in the cytoplasm. **(A–D’’)** Subcellular localization of Met-FLAG or 14-3-3 proteins in Kc cells after a 10 μM JHA treatment for 1 h. FLAG and HA antibodies were used for immunohistochemistry, DMSO was served as a negative control. **(E)** Effect of JHA on the expression level of 14-3-3ε and 14-3-3ζ at 2 h after the treatments. Kr-h1 was used a positive control. **p < 0.01 (Student’s t-test). **(F,G)** Effects of 14-3-3 overexpression on Kr-h1 mRNA level **(F)** and luciferase activity **(G)** driven by JHRR in Kc cells at 2 h after a 10 μM JHA treatment. Different letters indicate significant differences using one-way ANOVA followed by Duncan multiple comparisons test.

14-3-3 binding regulates the function of its targets through several mechanisms, such as by affecting protein complex stability and their subcellular localization (Tzivion and Avruch, 2002). We next investigated whether 14-3-3 proteins affect Met nuclear import and consequently JH action. In Kc cells, 14-3-3 proteins prevented Met nuclear import regardless of JHA treatment (Figures 7C–D’’, compared to Figures 7A–B’’). Meanwhile, JHA treatment significantly increased *Kr-h1* expression, whereas 14-3-3 transcript levels remained unchanged (Figure 7E), suggesting that JH evokes an antagonistic 14-3-3 response at the protein level. Moreover, overexpression of 14-3-3 proteins decreased *Kr-h1* expression or JH response region (JHRR) binding activity in Kc cells exposed to JHA (Figures 7F, G). Taken together, these results demonstrate that 14-3-3 proteins play a conserved role in the negative regulation of JH signal transduction by antagonizing Met nuclear import in an Hsp83-dependent manner.

### 3.7 JH-Induced Hsp83 Phosphorylation Promotes Hsp83-Met Binding Affinity

**FIGURE 8:**
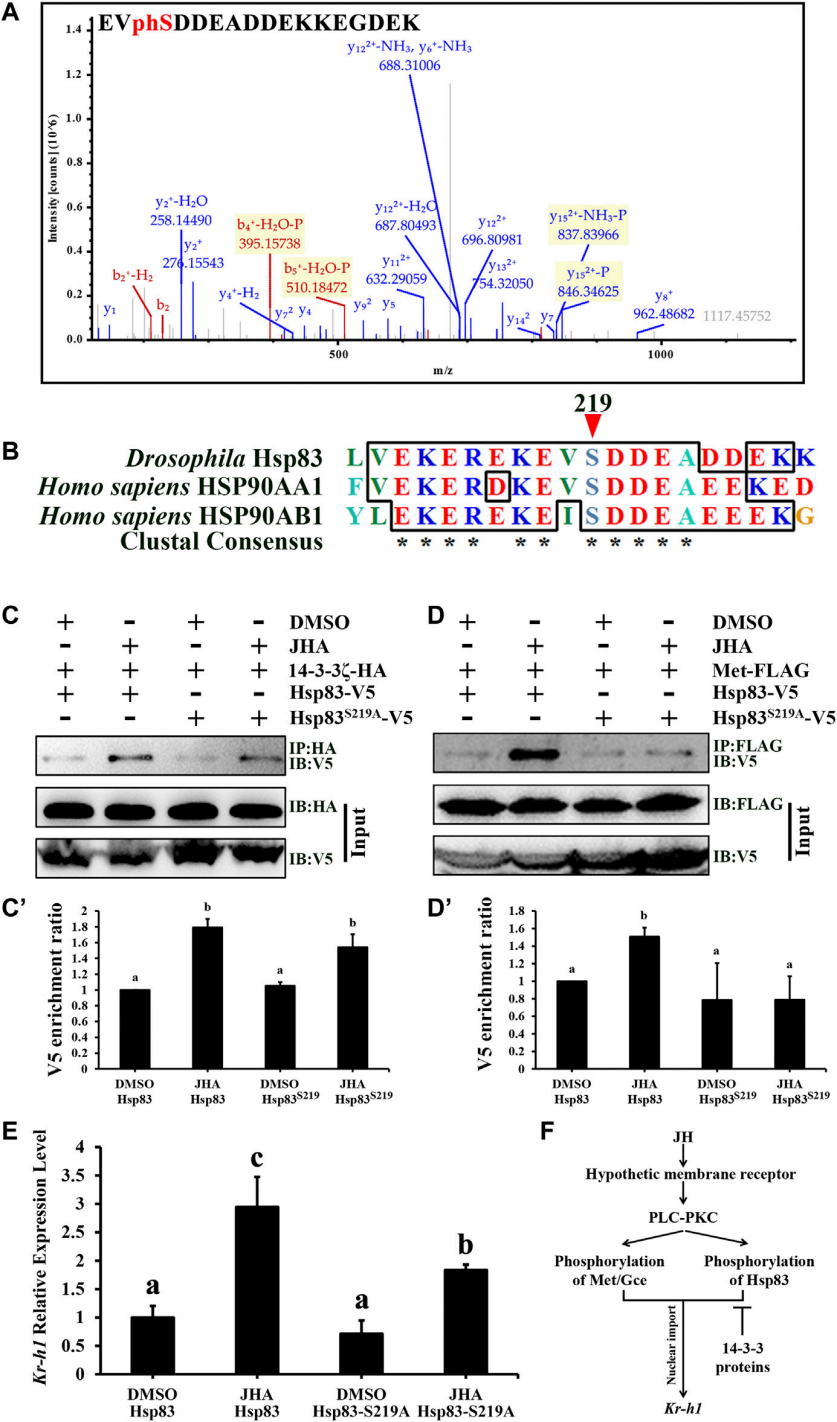
Phosphorylation of Hsp83 induced by JH increases its interaction with Met. **(A)** Phosphorylated S219 of Hsp83 identified from the JHA treated Kc cells, as determined by LC-MS/MS analysis. **(B)** Multiple alignment of Drosophila Hsp83 and its orthologs in the human. The phosphorylation site of Drosophila Hsp83 at S219 is marked with a red arrow. **(D)** melanogaster Hsp83: AAF47734, **(H)** sapiens Hsp90AA1: NP_001017963.2, and **(H)** sapiens Hsp90AB1: NP_001258898.1. **(C,D)** Kc cells were co-transfected with Act5c-Gal4, UAS-14-3-3ζ-HA **(C)** or UAS-Met-FLAG **(D)** and UAS-Hsp83-V5 or UAS-Hsp83S219A-V5 for 46 h, and treated with 1 μM JHA or DMSO for 2 h, and then cell lysates were subjected to immunoprecipitation (IP) with an anti-HA **(C)** or anti-FLAG **(D)** antibody, respectively. The interacting proteins were detected by immunoblot (IB). **(C’,D’)** Qualification of V5 of the western blotting results in **(C,D)** using tubulin as a control. **(E)** Downregulation of Kr-h1 in the presence of JHA by mutation of S219 in Hsp83. **(F)** A model showing that JH membrane signaling facilitates its intracellular signaling by phosphorylating Met/Gce or Hsp83, and 14-3-3 functions as a negative regulator of Met nuclear import. For panels **(C’)**, **(D’)**, and **(E)**, different letters indicate significant differences using one-way ANOVA followed by Duncan multiple comparisons test.

Normally, 14-3-3 proteins are characterized by their ability to bind target proteins through recognition of phosphorylated consensus motifs. Therefore, we speculated that Hsp83 is phosphorylated under JH regulation. As revealed by phosphoproteomics analysis, Hsp83 S219 was identified as a single phosphorylation site in the presence of JHA (Figure 8A), suggesting that JH induces Hsp83 phosphorylation and that 14-3-3 proteins might interact with Hsp83 in a phosphorylation-independent manner. Interestingly, as determined by aligning the Hsp83 protein of *D. melanogaster* Hsp83: AAF47734, *Homo sapiens* Hsp90AA1: NP_001017963.2, and *H. sapiens* Hsp90AB1: NP_001258898.1, it appears that phosphorylation of S219 appears to be highly conserved across *Drosophila* and other taxa (Figure 8B). To study whether S219 phosphorylation in Hsp83 is crucial for Met nuclear import, site-directed mutagenesis was performed to replace serine with alanine. S219A mutation significantly reduced the binding affinity of Met for Hsp83 but not for 14-3-3ζ under JHA treatment (Figures 8C–D’). This finding demonstrates that phosphorylation of Hsp83 at S219 promotes the interaction of Hsp83 with Met and further confirms that 14-3-3 proteins bind to Hsp83 in a phosphorylation-independent manner. Consequently, the S219A mutation attenuated *Kr-h1* transcription in response to JHA (Figure 8E). These results suggest that JH-induced Hsp83 phosphorylation at S219 facilitates the interaction between Hsp83 and Met to enhance JH intracellular signaling.

## 4 Discussion

In the present study, we adopted an efficient strategy for the expression and isolation of modified Met, Gce and their conjugates from *Drosophila* Kc cells. The system relies on the *in vitro* expression of the *E. coli* BirA enzyme as a fusion protein with Met/Gce that bears a short biotinylatable motif at the C-terminus. To increase the biotinylation efficiency, we took advantage of the self-cleaving activity of P2A peptides to digest tagged Met/Gce and the BirA enzyme, which were present in the same cellular microenvironment. Because of the strength and specificity of the streptavidin-biotin interaction, we were able to effectively isolate and enrich Met/Gce and their interacting proteins from Kc cells. Biotinylation with biotin derivatives was followed by purification on streptavidin resin (Rybak et al., 2004). This approach has been shown to be successful in recovering modified or bound proteins both *in vitro* and *in vivo* in *Drosophila* (Strübbe et al., 2011; Tykvart et al., 2012; Ramirez et al., 2015). This approach allowed us to identify phosphorylation sites of Met/Gce and those proteins that are directly or indirectly bound to Met/Gce by LC-MS/MS, then verify the findings by western blotting. Consistent with a previous study showing that JH membrane signaling induces phosphorylation of Met (Liu et al., 2015), we here further identified several phosphorylation sites of Met/Gce associated with JHA treatment. In *H. armigera* and *A. aegypti*, distinct phosphorylation sites of Met have been identified through phospho-antibody recognition or tag antibody purification. Interestingly, the phosphorylated equivalent residues were not detected in our results. A possible explanation is that Met phosphorylation at various sites may confer different molecular functions.

In *Drosophila*, nuclear import of JH receptors is essential for JH signal transduction (He et al., 2014). Although 4 JH-induced phosphorylation sites of Met were detected in the PAS-B+ domain, they seemed to be unimportant for Met nuclear import. With regard to the regulation of Met subcellular localization, the JH-dependent nuclear localization signal in the PAS-B+ domain may outweigh phosphorylation modification (Greb-Markiewicz et al., 2011). Previous studies have shown that the JH-dependent nuclear import of the PAS-B+ domain of Met is responsible for binding of the nuclear receptor Ftz-F1 (Bernardo and Dubrovsky, 2012; Kolonko et al., 2020). Conceivably, loss of phosphorylation modification in the PAS-B+ domain of Met could have no effect on JH intracellular signaling activity in *Drosophila*, differing from the report that T393 phosphorylation in the PAS-B domain is critical for *Kr-h1* transcription in *H. armigera* (Li et al., 2021). In our study, the *Kr-h1* transcription level was not affected when all 4 JH-induced phosphorylation sites were simultaneously mutated, indicating that these sites are dispensable for JH signal transduction in *Drosophila*. In contrast, the phosphorylation of S55 and T76 supported the DNA-binding function of the bHLH domain and maintained the normal expression of *Kr-h1*.

We further applied our strategy to *Drosophila* Kc cells *in vitro* and identified 14-3-3 proteins as important factors for nuclear import of Met in the presence of JHA. However, Tai was not detected in protein extracts from either the control or JHA-treated Kc cells. In addition, 14-3-3 proteins are identified to bind to JHRR isolated from JHA- or DMSO-treated Kc cells (He et al., 2014). In *Drosophila*, 14-3-3 proteins are encoded by two genes, *14-3-3ɛ* and *14-3-3ζ*. As conserved members of the phosphor-S/T binding protein family, 14-3-3ε and 14-3-3ζ form heterodimers to bind proteins containing phosphor-S/T-containing motifs via highly conserved amino acids housed in the active groove (Muslin et al., 1996; Yaffe and Elia, 2001). However, neither Met nor Gce contains these 14-3-3 binding motifs. Precipitation of 14-3-3 proteins could recover Met/Gce only in the presence of Hsp83 co-expressed with Met/Gce, indicating that Hsp83 is capable of bridging an interaction between Met/Gce and 14-3-3 proteins. Furthermore, we found that Hsp83 showed somewhat binding to all the 14-3-3 isoforms even in the absence of exogenous JH, suggesting that this interaction is likely to be widespread in other JH-independent signal transduction pathways.

In our study, only Hsp83 phosphorylated S219 was detected in JH-treated cells. A recent study reports that the phosphorylation level of Hsp83 at S219 is significantly higher in wild-type cells than in *Aug21* > *Grim* (JH-deficient) and *Met* ^*27*^ *gce* ^*2.5K*^ (lacking JH intracellular receptors) cells (Gao et al., 2021). This finding suggests that phosphorylation of Hsp83 at S219 is regulated primarily by JH. Bioinformatics analyses using the NetPhos 3.1 server (Blom et al., 1999) predicted that the S219 residue is susceptible to phosphorylation by casein kinase 2 (CK II). Given that CK II catalytic activity can be regulated by PKC, it is possible that S219 is phosphorylated by CK II through the JH-PLC-PKC pathway (Lee et al., 2016). Importantly, many previous studies have shown that CK II phosphorylates S231 and S226 in human Hsp90α and Hsp90β (Lees-Miller and Anderson, 1989), the equivalent residues have high homology with *Drosophila* Hsp83, respectively, that are essential for chemoresistance in leukemias or for the formation of the functional cytosolic aryl hydrocarbon receptor complex (Ogiso et al., 2004; Kurokawa et al., 2008).

In conclusion, this study reveals novel effects of JH membrane signaling on the transduction of JH intercellular signaling. On the one hand, JH membrane signaling potentiates the DNA-binding activity of the bHLH domain of Met. On the other hand, JH membrane signaling promotes the interaction between Hsp83 and Met by phosphorylating Hsp83 at S219. Finally, phosphorylation of Hsp83 modulates the JH response to enhance the transcriptional activity of Met, whereas 14-3-3 proteins negatively regulate JH action by physically interacting with Hsp83 (Figure 8F). This study contributes to our understanding of the complex JH signaling network.

## Data Availability

The datasets presented in this study can be found in online repositories. The mass spectrometry proteomics data have been deposited to the ProteomeXchange Consortium (http://proteomecentral.proteomexchange.org) *via* the iProX partner repository (Ma et al., 2019) with the dataset identifier PXD027314.

## Supplementary Material

Supplementary Figure 1 Subcellular localization of distinct fragments of Gce with a FLAG tag in Kc cells. **(A)** Scheme illustrating the full-length and truncated mutants of Gce. **(B–G’)** Localization of truncated mutants of Gce was not altered in the absence or presence of JHA. Cells expressing truncated mutants of Gce were stained for FLAG (green). Merge images also present nuclear staining with DAPI (blue).

## References

[B1] Abdou M. A. He Q. Wen D. Zyaan O. Wang J. Xu J. (2011). *Drosophila* Met and Gce Are Partially Redundant in Transducing Juvenile Hormone Action. Insect Biochem. Mol. Biol. 41, 938–945. 10.1016/j.ibmb.2011.09.003 21968404

[B2] Acevedo S. F. Tsigkari K. K. Grammenoudi S. Skoulakis E. M. C. (2007). *In Vivo* functional Specificity and Homeostasis of Drosophila 14-3-3 Proteins. Genetics 177, 239–253. 10.1534/genetics.107.072280 17660572 PMC2013677

[B3] Ashok M. Turner C. Wilson T. G. (1998). Insect Juvenile Hormone Resistance Gene Homology with the bHLH-PAS Family of Transcriptional Regulators. Proc. Natl. Acad. Sci. U.S.A. 95, 2761–2766. 10.1073/pnas.95.6.2761 9501163 PMC19642

[B4] Bai H. Palli S. R. (2016). Identification of G Protein-Coupled Receptors Required for Vitellogenin Uptake into the Oocytes of the Red Flour Beetle, *Tribolium castaneum* . Sci. Rep. 6, 27648. 10.1038/srep27648 27277501 PMC4899757

[B5] Baumann A. Barry J. Wang S. Fujiwara Y. Wilson T. G. (2010). Paralogous Genes Involved in Juvenile Hormone Action in *Drosophila melanogaster* . Genetics 185, 1327–1336. 10.1534/genetics.110.116962 20498297 PMC2927759

[B6] Beckett D. Kovaleva E. Schatz P. J. (1999). A Minimal Peptide Substrate in Biotin Holoenzyme Synthetase-Catalyzed Biotinylation. Protein Sci. 8, 921–929. 10.1110/ps.8.4.921 10211839 PMC2144313

[B7] Benton R. Johnston D. S. (2003). *Drosophila* PAR-1 and 14-3-3 Inhibit Bazooka/PAR-3 to Establish Complementary Cortical Domains in Polarized Cells. Cell 115, 691–704. 10.1016/s0092-8674(03)00938-3 14675534

[B8] Bernardo T. J. Dubrovsky E. B. (2012). The *Drosophila* Juvenile Hormone Receptor Candidates Methoprene-Tolerant (MET) and Germ Cell-Expressed (GCE) Utilize a Conserved LIXXL Motif to Bind the FTZ-F1 Nuclear Receptor. J. Biol. Chem. 287, 7821–7833. 10.1074/jbc.m111.327254 22249180 PMC3293574

[B9] Blom N. Gammeltoft S. Brunak S. (1999). Sequence and Structure-Based Prediction of Eukaryotic Protein Phosphorylation Sites. J. Mol. Biol. 294, 1351–1362. 10.1006/jmbi.1999.3310 10600390

[B10] Charles J.-P. Iwema T. Epa V. C. Takaki K. Rynes J. Jindra M. (2011). Ligand-binding Properties of a Juvenile Hormone Receptor, Methoprene-Tolerant. Proc. Natl. Acad. Sci. U.S.A. 108, 21128–21133. 10.1073/pnas.1116123109 22167806 PMC3248530

[B11] Daniels R. W. Rossano A. J. Macleod G. T. Ganetzky B. (2014). Expression of Multiple Transgenes from a Single Construct Using Viral 2A Peptides in *Drosophila* . PLoS One 9, e100637. 10.1371/journal.pone.0100637 24945148 PMC4063965

[B12] Davey K. G. Huebner E. (1974). The Response of the Follicle Cells of *Rhodnius prolixus* to Juvenile Hormone and Antigonadotropin *In Vitro* . Can. J. Zool. 52, 1407–1412. 10.1139/z74-178 4611604

[B13] Davey K. G. (2000). The Modes of Action of Juvenile Hormones: Some Questions We Ought to Ask. Insect Biochem. Mol. Biol. 30, 663–669. 10.1016/s0965-1748(00)00037-0 10876109

[B14] De Boer E. Rodriguez P. Bonte E. Krijgsveld J. Katsantoni E. Heck A. (2003). Efficient Biotinylation and Single-step Purification of Tagged Transcription Factors in Mammalian Cells and Transgenic Mice. Proc. Natl. Acad. Sci. U.S.A. 100, 7480–7485. 10.1073/pnas.1332608100 12802011 PMC164612

[B15] Dougherty M. K. Morrison D. K. (2004). Unlocking the Code of 14-3-3. J. Cel Sci. 117, 1875–1884. 10.1242/jcs.01171 15090593

[B16] Gao Y. Liu S. Jia Q. Wu L. Yuan D. Li E. Y. Feng Q. Wang G. Palli S. R. Wang J. Li S. (2021). Juvenile Hormone Membrane Signaling Phosphorylates USP and Thus Potentiates 20-hydroxyecdysone Action in Drosophila. Sci. Bull. 67, 186–197. 10.1016/j.scib.2021.06.019 36546012

[B17] Godlewski J. Wang S. Wilson T. G. (2006). Interaction of bHLH-PAS Proteins Involved in Juvenile Hormone Reception in *Drosophila* . Biochem. Biophysical Res. Commun. 342, 1305–1311. 10.1016/j.bbrc.2006.02.097 16516852

[B18] Greb-Markiewicz B. Orłowski M. Dobrucki J. Ożyhar A. (2011). Sequences that Direct Subcellular Traffic of the *Drosophila* Methoprene-Tolerant Protein (MET) Are Located Predominantly in the PAS Domains. Mol. Cell Endocrinol. 345, 16–26. 10.1016/j.mce.2011.06.035 21745535

[B19] He Q. Wen D. Jia Q. Cui C. Wang J. Palli S. R. (2014). Heat Shock Protein 83 (Hsp83) Facilitates Methoprene-Tolerant (Met) Nuclear Import to Modulate Juvenile Hormone Signaling. J. Biol. Chem. 289, 27874–27885. 10.1074/jbc.m114.582825 25122763 PMC4183821

[B20] He Q. Zhang Y. Zhang X. Xu D. Dong W. Li S. (2017). Nucleoporin Nup358 Facilitates Nuclear Import of Methoprene-Tolerant (Met) in an Importin β- and Hsp83-dependent Manner. Insect Biochem. Mol. Biol. 81, 10–18. 10.1016/j.ibmb.2016.12.005 27979731

[B21] Hunter T. Karin M. (1992). The Regulation of Transcription by Phosphorylation. Cell 70, 375–387. 10.1016/0092-8674(92)90162-6 1643656

[B22] Ji H.-H. Ostap E. M. (2020). The Regulatory Protein 14-3-3β Binds to the IQ Motifs of Myosin-IC Independent of Phosphorylation. J. Biol. Chem. 295, 3749–3756. 10.1074/jbc.ra119.011227 31811090 PMC7086031

[B23] Jindra M. Bellés X. Shinoda T. (2015a). Molecular Basis of Juvenile Hormone Signaling. Curr. Opin. Insect Sci. 11, 39–46. 10.1016/j.cois.2015.08.004 28285758

[B24] Jindra M. Uhlirova M. Charles J.-P. Smykal V. Hill R. J. (2015b). Genetic Evidence for Function of the bHLH-PAS Protein Gce/Met as a Juvenile Hormone Receptor. Plos Genet. 11, e1005394. 10.1371/journal.pgen.1005394 26161662 PMC4498814

[B25] Kayukawa T. Minakuchi C. Namiki T. Togawa T. Yoshiyama M. Kamimura M. (2012). Transcriptional Regulation of Juvenile Hormone-Mediated Induction of Krüppel Homolog 1, a Repressor of Insect Metamorphosis. Proc. Natl. Acad. Sci. U.S.A. 109, 11729–11734. 10.1073/pnas.1204951109 22753472 PMC3406821

[B26] Kim K. Albishi N. M. Palli S. R. (2021). Identification of Juvenile Hormone-Induced Posttranslational Modifications of Methoprene Tolerant and Krüppel Homolog 1 in the Yellow Fever Mosquito, *Aedes aegypti* . J. Proteomics 242, 104257. 10.1016/j.jprot.2021.104257 33957312 PMC8218339

[B27] Kolonko M. Bystranowska D. Taube M. Kozak M. Bostock M. Popowicz G. (2020). The Intrinsically Disordered Region of GCE Protein Adopts a More Fixed Structure by Interacting with the LBD of the Nuclear Receptor FTZ-F1. Cell Commun. Signal. 18, 180. 10.1186/s12964-020-00662-2 33153474 PMC7643343

[B28] Kurokawa M. Zhao C. Reya T. Kornbluth S. (2008). Inhibition of Apoptosome Formation by Suppression of Hsp90β Phosphorylation in Tyrosine Kinase-Induced Leukemias. Mol. Cel Biol. 28, 5494–5506. 10.1128/mcb.00265-08 PMC2519729 18591256

[B29] Lee Y.-H. Park J.-W. Bae Y.-S. (2016). Regulation of Protein Kinase CK2 Catalytic Activity by Protein Kinase C and Phospholipase D2. Biochimie 121, 131–139. 10.1016/j.biochi.2015.12.005 26703243

[B30] Lees-Miller S. P. Anderson C. W. (1989). Two Human 90-kDa Heat Shock Proteins Are Phosphorylated *In Vivo* at Conserved Serines that Are Phosphorylated *In Vitro* by Casein Kinase II. J. Biol. Chem. 264, 2431–2437. 10.1016/s0021-9258(19)81631-9 2492519

[B31] Li M. Mead E. A. Zhu J. (2011). Heterodimer of Two bHLH-PAS Proteins Mediates Juvenile Hormone-Induced Gene Expression. Proc. Natl. Acad. Sci. U.S.A. 108, 638–643. 10.1073/pnas.1013914108 21187375 PMC3021087

[B32] Li X. Duan X. Jiang H. Sun Y. Tang Y. Yuan Z. (2006). Genome-wide Analysis of basic/helix-loop-helix Transcription Factor Family in rice and Arabidopsis. Plant Physiol. 141, 1167–1184. 10.1104/pp.106.080580 16896230 PMC1533929

[B33] Li Y.-X. Wang D. Zhao W.-L. Zhang J.-Y. Kang X.-L. Li Y.-L. (2021). Juvenile Hormone Induces Methoprene-Tolerant 1 Phosphorylation to Increase Interaction with Taiman in *Helicoverpa Armigera* . Insect Biochem. Mol. Biol. 130, 103519. 10.1016/j.ibmb.2021.103519 33450383

[B34] Liu P. Peng H. J. Zhu J. (2015). Juvenile Hormone-Activated Phospholipase C Pathway Enhances Transcriptional Activation by the Methoprene-Tolerant Protein. Proc. Natl. Acad. Sci. U. S. A. 112, E1871–E1879. 10.1073/pnas.1423204112 25825754 PMC4403202

[B35] Liu S. Li K. Gao Y. Liu X. Chen W. Ge W. (2018). Antagonistic Actions of Juvenile Hormone and 20-hydroxyecdysone within the Ring Gland Determine Developmental Transitions in *Drosophila* . Proc. Natl. Acad. Sci. U.S.A. 115, 139–144. 10.1073/pnas.1716897115 29255055 PMC5776822

[B36] Liu Y. Sheng Z. Liu H. Wen D. He Q. Wang S. (2009). Juvenile Hormone Counteracts the bHLH-PAS Transcription Factors MET and GCE to Prevent Caspase-dependent Programmed Cell Death inDrosophila. Development 136, 2015–2025. 10.1242/dev.033712 19465595

[B37] Liu Z. Chen O. Wall J. B. J. Zheng M. Zhou Y. Wang L. (2017). Systematic Comparison of 2A Peptides for Cloning Multi-Genes in a Polycistronic Vector. Sci. Rep. 7, 2193. 10.1038/s41598-017-02460-2 28526819 PMC5438344

[B38] Ma J. Chen T. Wu S. Yang C. Bai M. Shu K. (2019). iProX: an Integrated Proteome Resource. Nucleic Acids Res. 47, D1211–D1217. 10.1093/nar/gky869 30252093 PMC6323926

[B39] Mackintosh C. (2004). Dynamic Interactions between 14-3-3 Proteins and Phosphoproteins Regulate Diverse Cellular Processes. Biochem. J. 381, 329–342. 10.1042/bj20031332 15167810 PMC1133837

[B40] Miura K. Oda M. Makita S. Chinzei Y. (2005). Characterization of the Drosophila Methoprene -tolerant Gene Product. FEBS J. 272, 1169–1178. 10.1111/j.1742-4658.2005.04552.x 15720391

[B41] Morrison D. K. (2009). The 14-3-3 Proteins: Integrators of Diverse Signaling Cues that Impact Cell Fate and Cancer Development. Trends Cel Biol. 19, 16–23. 10.1016/j.tcb.2008.10.003 PMC3073487 19027299

[B42] Muslin A. J. Tanner J. W. Allen P. M. Shaw A. S. (1996). Interaction of 14-3-3 with Signaling Proteins Is Mediated by the Recognition of Phosphoserine. Cell 84, 889–897. 10.1016/s0092-8674(00)81067-3 8601312

[B43] Ogiso H. Kagi N. Matsumoto E. Nishimoto M. Arai R. Shirouzu M. (2004). Phosphorylation Analysis of 90 kDa Heat Shock Protein within the Cytosolic Arylhydrocarbon Receptor Complex. Biochemistry 43, 15510–15519. 10.1021/bi048736m 15581363

[B44] Ojani R. Liu P. Fu X. Zhu J. (2016). Protein Kinase C Modulates Transcriptional Activation by the Juvenile Hormone Receptor Methoprene-Tolerant. Insect Biochem. Mol. Biol. 70, 44–52. 10.1016/j.ibmb.2015.12.001 26689644 PMC4767628

[B45] Ottmann C. Yasmin L. Weyand M. Veesenmeyer J. L. Diaz M. H. Palmer R. H. (2007). Phosphorylation-independent Interaction between 14-3-3 and Exoenzyme S: from Structure to Pathogenesis. EMBO J. 26, 902–913. 10.1038/sj.emboj.7601530 17235285 PMC1794388

[B46] Rajan S. Preisig‐Müller R. Wischmeyer E. Nehring R. Hanley P. J. Renigunta V. (2002). Interaction with 14‐3‐3 Proteins Promotes Functional Expression of the Potassium Channels TASK‐1 and TASK‐3. J. Physiol. 545, 13–26. 10.1113/jphysiol.2002.027052 12433946 PMC2290646

[B47] Ramirez J. Martinez A. Lectez B. Lee S. Y. Franco M. Barrio R. (2015). Proteomic Analysis of the Ubiquitin Landscape in the *Drosophila* Embryonic Nervous System and the Adult Photoreceptor Cells. PLoS One 10, e0139083. 10.1371/journal.pone.0139083 26460970 PMC4604154

[B48] Riddiford L. M. Truman J. W. Mirth C. K. Shen Y.-c. (2010). A Role for Juvenile Hormone in the Prepupal Development of *Drosophila melanogaster* . Development 137, 1117–1126. 10.1242/dev.037218 20181742 PMC2835327

[B49] Rybak J.-N. Scheurer S. B. Neri D. Elia G. (2004). Purification of Biotinylated Proteins on Streptavidin Resin: a Protocol for Quantitative Elution. Proteomics 4, 2296–2299. 10.1002/pmic.200300780 15274123

[B50] Strübbe G. Popp C. Schmidt A. Pauli A. Ringrose L. Beisel C. (2011). Polycomb Purification by *In Vivo* Biotinylation Tagging Reveals Cohesin and Trithorax Group Proteins as Interaction Partners. Proc. Natl. Acad. Sci. U. S. A. 108, 5572–5577. 10.1073/pnas.1007916108 21415365 PMC3078387

[B51] Tian L. Ma L. Guo E. Deng X. Ma S. Xia Q. (2013). 20-hydroxyecdysone upregulatesAtggenes to Induce Autophagy in the Bombyx Fat Body. Autophagy 9, 1172–1187. 10.4161/auto.24731 23674061 PMC3748190

[B52] Tykvart J. Šácha P. Bařinka C. Knedlík T. Starková J. Lubkowski J. (2012). Efficient and Versatile One-step Affinity Purification of *In Vivo* Biotinylated Proteins: Expression, Characterization and Structure Analysis of Recombinant Human Glutamate Carboxypeptidase II. Protein Expr. Purif. 82, 106–115. 10.1016/j.pep.2011.11.016 22178733 PMC3443621

[B53] Tzivion G. Avruch J. (2002). 14-3-3 Proteins: Active Cofactors in Cellular Regulation by Serine/threonine Phosphorylation. J. Biol. Chem. 277, 3061–3064. 10.1074/jbc.r100059200 11709560

[B54] Wilson T. G. Fabian J. (1986). A *Drosophila melanogaster* Mutant Resistant to a Chemical Analog of Juvenile Hormone. Dev. Biol. 118, 190–201. 10.1016/0012-1606(86)90087-4 3095161

[B55] Yaffe M. B. Elia A. E. H. (2001). Phosphoserine/threonine-binding Domains. Curr. Opin. Cel Biol. 13, 131–138. 10.1016/s0955-0674(00)00189-7 11248545

[B56] Yamamoto K. Chadarevian A. Pellegrini M. (1988). Juvenile Hormone Action Mediated in Male Accessory Glands of Drosophila by Calcium and Kinase C. Science 239, 916–919. 10.1126/science.3124270 3124270

[B57] Zhang Z. Xu J. Sheng Z. Sui Y. Palli S. R. (2011). Steroid Receptor Co-activator Is Required for Juvenile Hormone Signal Transduction through a bHLH-PAS Transcription Factor, Methoprene Tolerant. J. Biol. Chem. 286, 8437–8447. 10.1074/jbc.m110.191684 21190938 PMC3048728

