# Supplementary figures and images for: Juvenile Hormone Membrane Signaling Enhances its Intracellular Signaling Through Phosphorylation of Met and Hsp83
Source: Front Physiol. 2022 Apr 27;13:872889. doi: 10.3389/fphys.2022.872889 (PMC9091338; doi:10.3389/fphys.2022.872889)

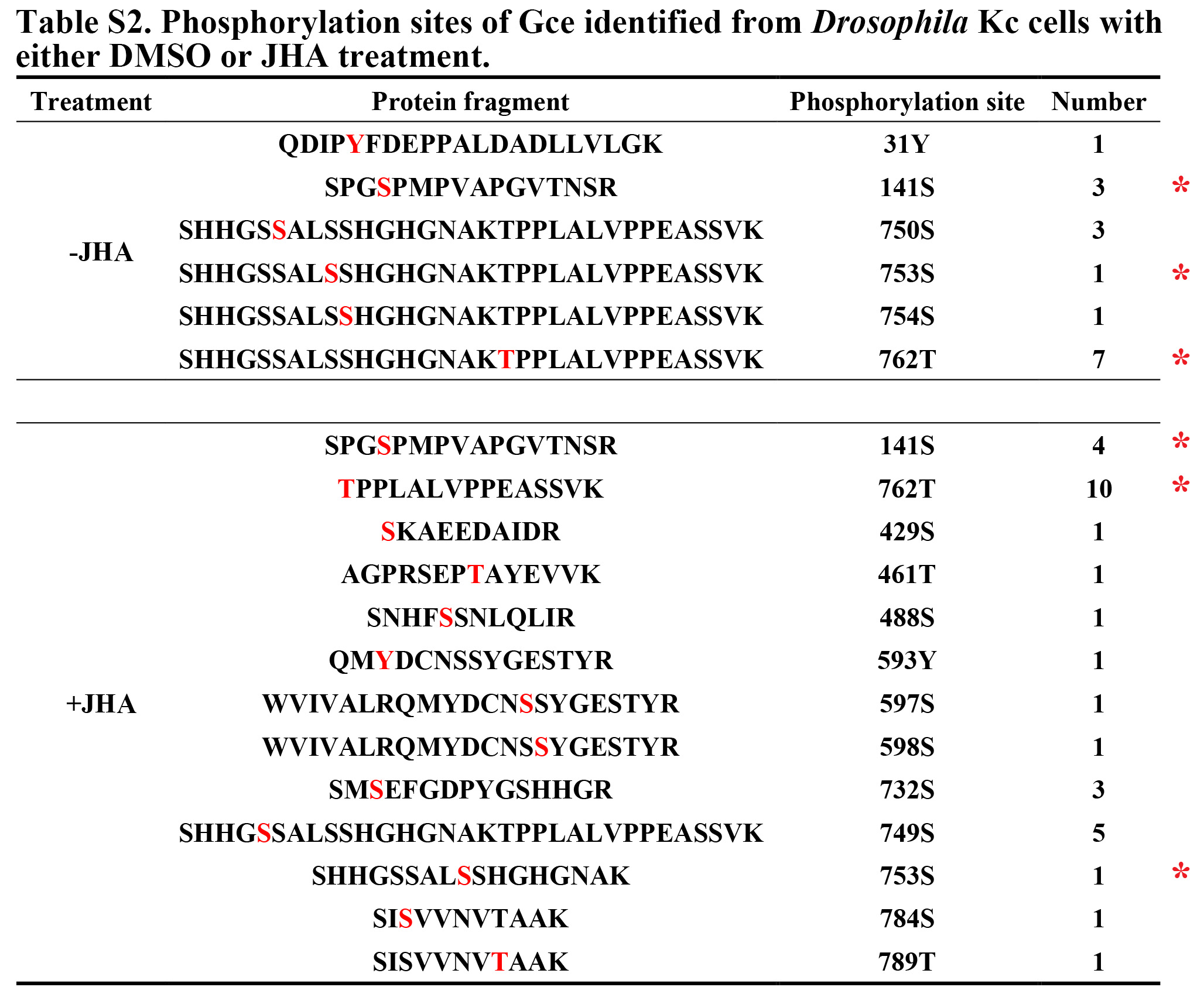

Supplement: Supplementary file 2 [file Image3.JPEG]

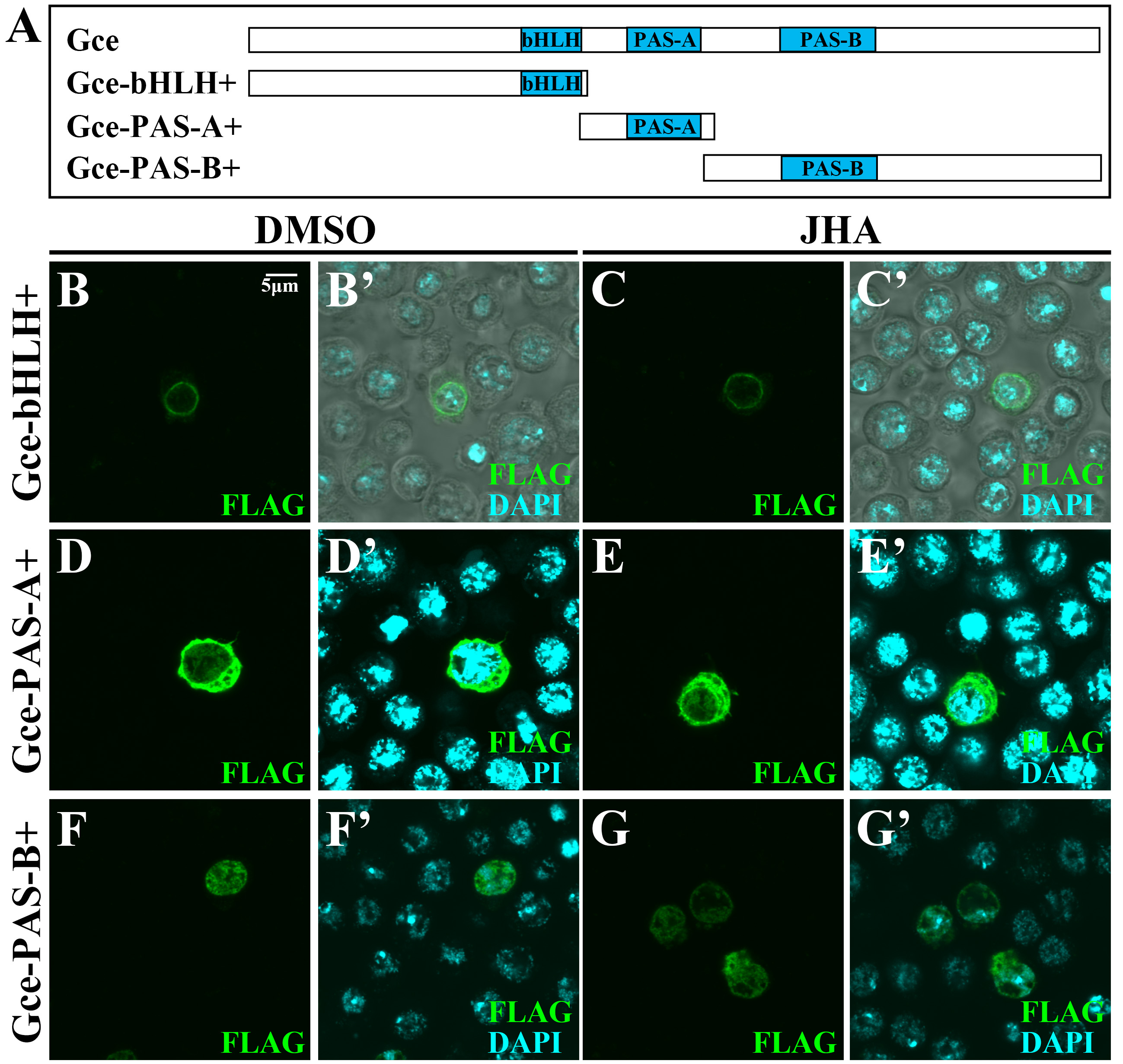

Supplement: Supplementary file 5 [file Image1.JPEG]

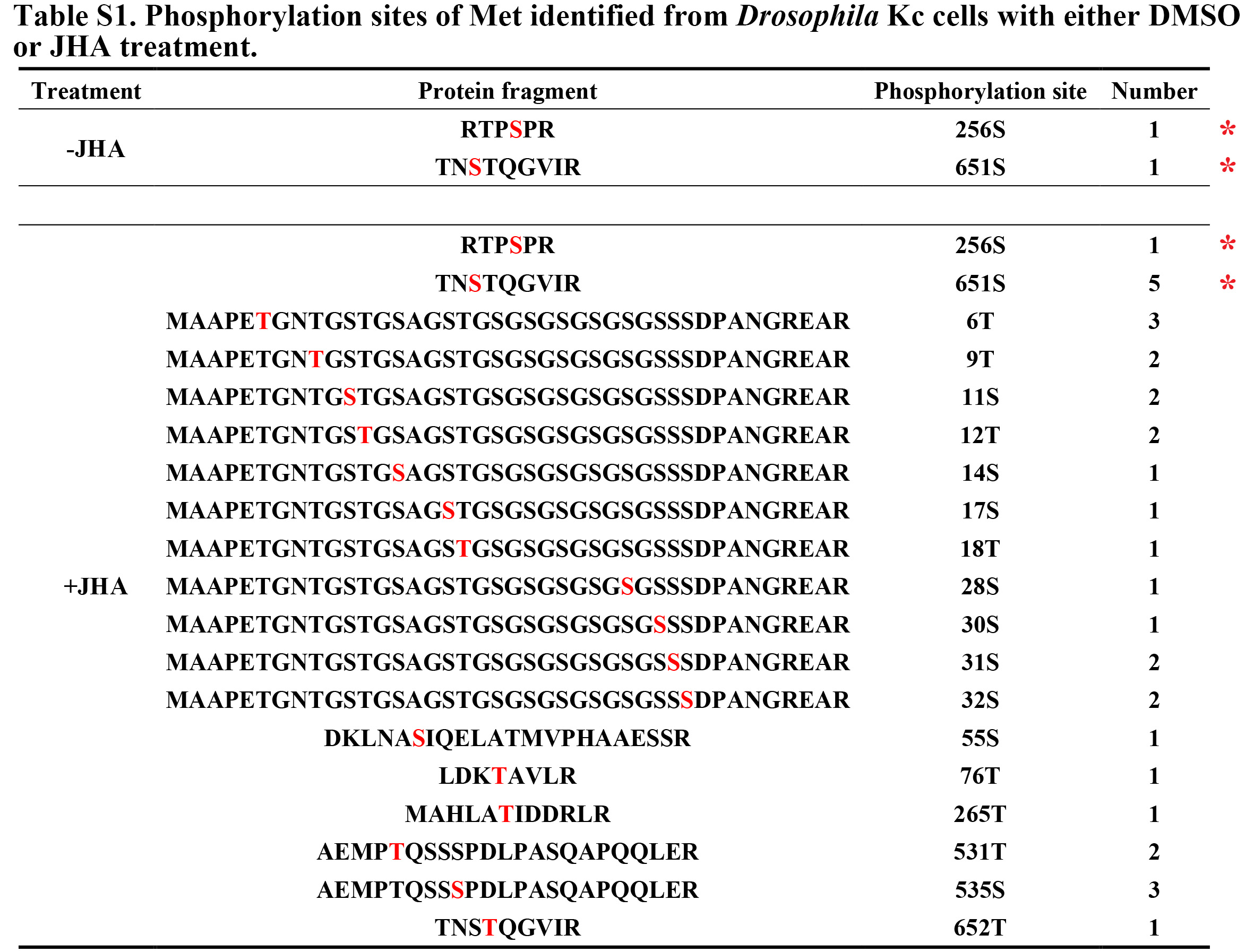

Supplement: Supplementary file 7 [file Image2.JPEG]
