# Supplementary material for: Juvenile Hormone Membrane Signaling Enhances its Intracellular Signaling Through Phosphorylation of Met and Hsp83
Source: Front Physiol. 2022 Apr 27;13:872889. doi: 10.3389/fphys.2022.872889 (PMC9091338; doi:10.3389/fphys.2022.872889)
Supplement: Supplementary file 8 [file Table3.DOCX]

| **Protein IDs** | **Protein names** | **Biological Process** | **Molecular Function** |
| --- | --- | --- | --- |
| P92177 | 14-3-3epsilon | Regulation of signal transduction | Phosphoserine residue binding |
| A0A0B4KEH0;P29310 | 14-3-3zeta | Regulation of signal transduction | Phosphoserine residue binding |
| Q9VLC5 | Aldh | acetaldehyde metabolic process | Acetaldehyde dehydrogenase activity |
| X2JH42;L0MQ04;Q05825;Q8T4C4 | ATPsynbeta; ATPsyn-beta | ATP synthesis coupled proton transport | ATPase activity |
| A4V303;P48605 | Cctgamma | Protein folding | ATPase activity |
| Q9W3C0 | CG1789 | rRNA processing | NA |
| Q7KW39 | CG17896 | oxidation-reduction process | Malonate-semialdehyde dehydrogenase activity |
| X2JDP6;Q8T079;M9MRG2 | CG4747 | Positive regulation of histone acetylation | Chromatin binding |
| A1Z6M6 | Dpit47 | NA | Hsp90 protein binding |
| Q9VFV9 | Droj2 | Protein folding | Heat shock protein binding |
| Q8IPB1;Q9V3I1 | dUTPase | dUTP metabolic process | dUTP diphosphatase activity |
| P08736;A4V3Q6;P05303 | Ef1alpha48D; Ef1alpha100E | Translational elongation | Translation elongation factor activity |
| A0A0B4LFL3;O96827 | Ef1beta | Translational elongation | Translation elongation factor activity |
| A0A0B4JD11;Q9NJH0 | Ef1gamma | Translational elongation | Translation elongation factor activity |
| X2JFR6;P41374 | eIF-2alpha | Translational initiation | Translation initiation factor activity |
| Q7JXZ2 | Eip55E | Glutathione biosynthetic process | Carbon-sulfur lyase activity |
| Q3YMU0 | ERp60 | cell redox homeostasis | Protein disulfide isomerase activity |
| M9PJN8;P07487;P07486 | Gapdh2; Gapdh1 | Glucose homeostasis | Glyceraldehyde-3-phosphate dehydrogenase (NAD+) activity |
| Q9VJD1 | GCS2beta | N-glycan processing | NA |
| P54385;A0A0C4DHE7;Q8IMY1 | Gdh | glutamate metabolic process | Glutamate dehydrogenase activity |
| Q9V3D8;A0A0C4FEI8 | grsm | Proteolysis | Metalloaminopeptidase activity |
| P02283 | His2B | NA | Protein heterodimerization activity |
| Q9VPN5 | Hop | NA | Hsp90 protein binding |
| E1JHA4;P48809 | Hrb27C | Regulation of translation and mRNA splicing | Translation repressor activity |
| Q9VUC1;M9MSL3 | Hsc70Cb | Chaperone-mediated protein complex assembly | NA |
| O02649 | Hsp60 | Protein folding | Chaperone binding |
| O97125;Q9VG58;Q9BIS2;Q9BIR7;Q8INI8;P82910;P02825;P11146 | Hsp68; Hsp70Bbb; Hsp70Bb; Hsp70Bc; Hsp70Ba; Hsp70Aa; Hsp70Ab; Hsc70-2 | Protein refolding | Protein folding chaperone |
| Q7KUB0;Q8IQA7;Q9VSI6;Q7KUB1;B7Z0E0 | Idh | Isocitrate metabolic process | Isocitrate dehydrogenase activity |
| B5RJQ0;Q95028 | ImpL3 | Carbohydrate metabolic process | L-lactate dehydrogenase activity |
| X2JF59;Q9V3P0 | Jafrac1 | Cell redox homeostasis | Thioredoxin peroxidase activity |
| A0A023GQA5;P24156 | l(2)37Cc | Mitochondrion organization | NA |
| M9NE89;P08928 | Lam | Positive regulation of cytokinesis, actomyosin contractile ring assembly | Protein binding |
| A0A0B4K8A5;Q9W2F2 | mahj | Protein ubiquitination | NA |
| A0A0B4K7G4;P13469 | mod | mRNA splicing, via spliceosome | Nucleic acid binding |
| A0A0B4LEY6;Q94518 | Nacalpha | Protein targeting to membrane | Protein binding |
| A0A0B4KI24;Q27415 | Nlp | Chromatin remodeling | Histone binding |
| Q0E924;A8DYI7;A8DYI6 | Phb2 | Mitochondrion organization | NA |
| M9PD75;Q94920 | porin | Anion transmembrane transport | Voltage-gated anion channel activity |
| E1JGZ9;Q9XZJ4 | Prosalpha1 | Proteasome-mediated ubiquitin-dependent protein catabolic process | Endopeptidase activity |
| O62619 | PyK | Glucose homeostasis | Pyruvate kinase activity |
| M9PCC1;O18640 | Rack1 | Positive regulation of protein phosphorylation | Protein-macromolecule adaptor activity |
| A4V4A5;Q9VZ23 | Ran;ran | Intracellular protein transport | GTPase activity |
| P48591 | RnrL | Deoxyribonucleotide biosynthetic process | Ribonucleoside-diphosphate reductase activity |
| Q24492 | RpA-70 | DNA replication | Single-stranded DNA binding |
| A0A0B4LGZ5;P46222 | RpL11 | Translation | Structural constituent of ribosome |
| Q9W1B9 | RpL12 | Translation | Structural constituent of ribosome |
| M9PFF0;P41126 | RpL13 | Translation | Structural constituent of ribosome |
| P50887 | RpL22 | Translation | Structural constituent of ribosome |
| Q9VVU2 | RpL26 | Translation | Structural constituent of ribosome |
| R9Q794;Q9W5R8 | RpL5 | Translation | Structural constituent of ribosome |
| M9PG76;P19889 | RpLP0 | Translation | Structural constituent of ribosome |
| Q9V3Z4 | Rpn5 | Proteasome-mediated ubiquitin-dependent protein catabolic process | NA |
| X2JCX8;C0HKA1;C0HKA0 | RpS14b | Translation | Structural constituent of ribosome |
| E1JJM9;P48149;Q7KR04 | RpS15Aa;RpS15Ab | Translation | Structural constituent of ribosome |
| A0A0B4LG52;Q9W237 | RpS16 | Translation | Structural constituent of ribosome |
| P17704 | RpS17 | Translation | Structural constituent of ribosome |
| P41094 | RpS18 | Translation | Structural constituent of ribosome |
| E2QD65;P39018 | RpS19a | Translation | Structural constituent of ribosome |
| Q9VBU9 | RpS27 | Translation | Structural constituent of ribosome |
| G3M3A2;Q06559 | RpS3 | Translation | Structural constituent of ribosome |
| P55830 | RpS3A | Translation | Structural constituent of ribosome |
| X2JKU5;Q24186;A0A0B4K683;Q9VFE4 | RpS5a; RpS5b | Translation | Structural constituent of ribosome |
| Q95TP9;P29327 | RpS6 | Translation | Structural constituent of ribosome |
| A0A0B4K6N1;Q8MLY8 | RpS8 | Translation | Structural constituent of ribosome |
| A0A0B4K6U6;Q08473 | sqd | Localization and translational regulation of mRNA | RNA binding |
| P38979 | sta | Translation | Structural constituent of ribosome |
| Q9VHL2 | Tcp-1eta | Protein folding | Unfolded protein binding |
| Q9VXQ5 | Tcp-1zeta | Protein folding | Unfolded protein binding |
| Q9VV75 | UQCR-C2 | Mitochondrial electron transport, ubiquinol to cytochrome c | Ubiquinol-cytochrome-c reductase activity |
